# Author Correction: Energy-saving and product-oriented hydrogen peroxide electrosynthesis enabled by electrochemistry pairing and product engineering

**DOI:** 10.1038/s41467-024-52281-x

**Published:** 2024-09-06

**Authors:** Jun Qi, Yadong Du, Qi Yang, Na Jiang, Jiachun Li, Yi Ma, Yangjun Ma, Xin Zhao, Jieshan Qiu

**Affiliations:** State Key Laboratory of Chemical Resource Engineering, College of Chemical Engineering, Beijing University of Chemical Technology, Beijing, 100029 P. R. China

Correction to: *Nature Communications* 10.1038/s41467-023-41997-x, published online 7 October 2023

The original version of the Supplementary Information for this manuscript contained an error in Supplementary Fig. 19a, which plotted identical Raman data at applied potentials of 1.2 V and 1.3 V during the in-situ analysis of ethylene glycol electrooxidation. The corrected data are provided in the new Supplementary Information file and the Source Data have been updated to reflect the correct data at the different applied potentials. A comparison of Supplementary Fig. 19a before and after correction is shown below.
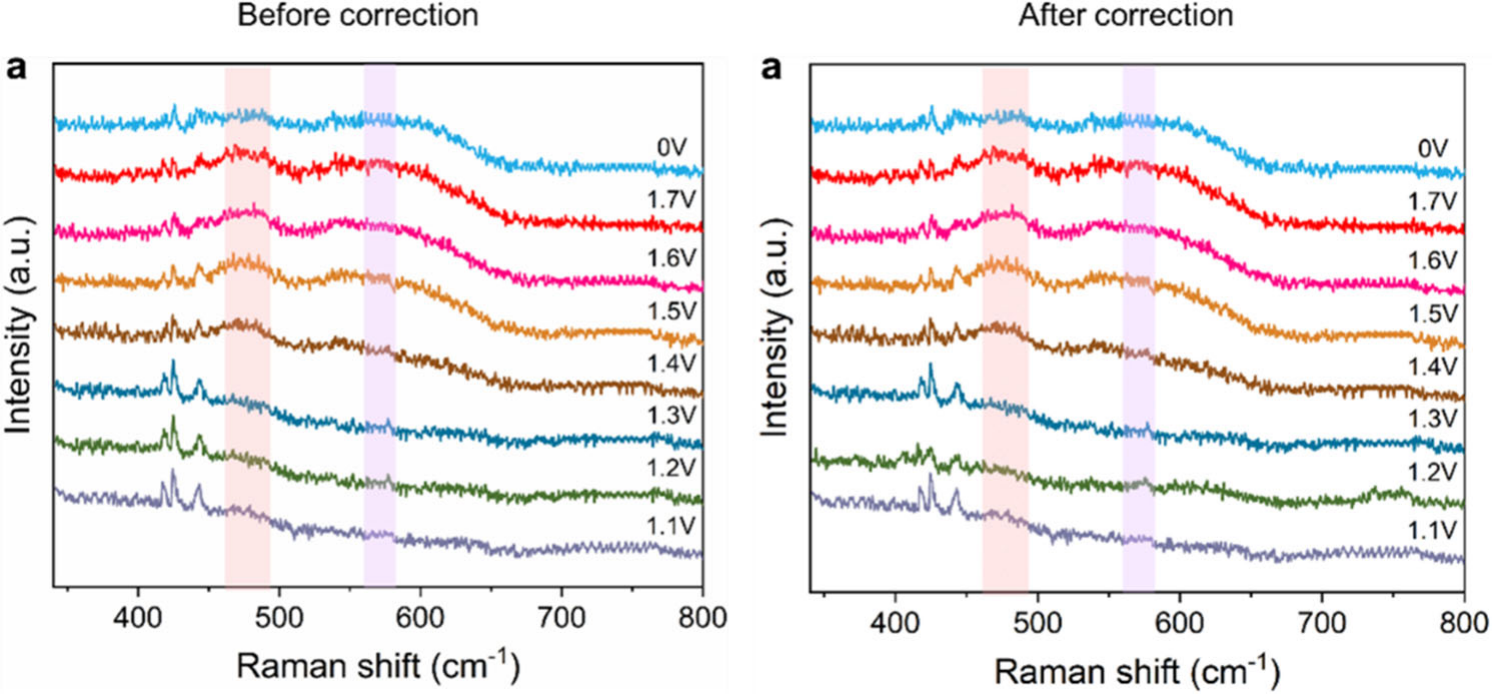


The original version of the Supplementary Information for this manuscript contained a non-technical error in Supplementary Fig. 11a. The corrected data for **Ni1Mn1-MOF-Se/NF** are provided in the new Supplementary Information file and the Source Data have been updated to reflect this change. A comparison of Supplementary Fig. 11a before and after correction is shown below.
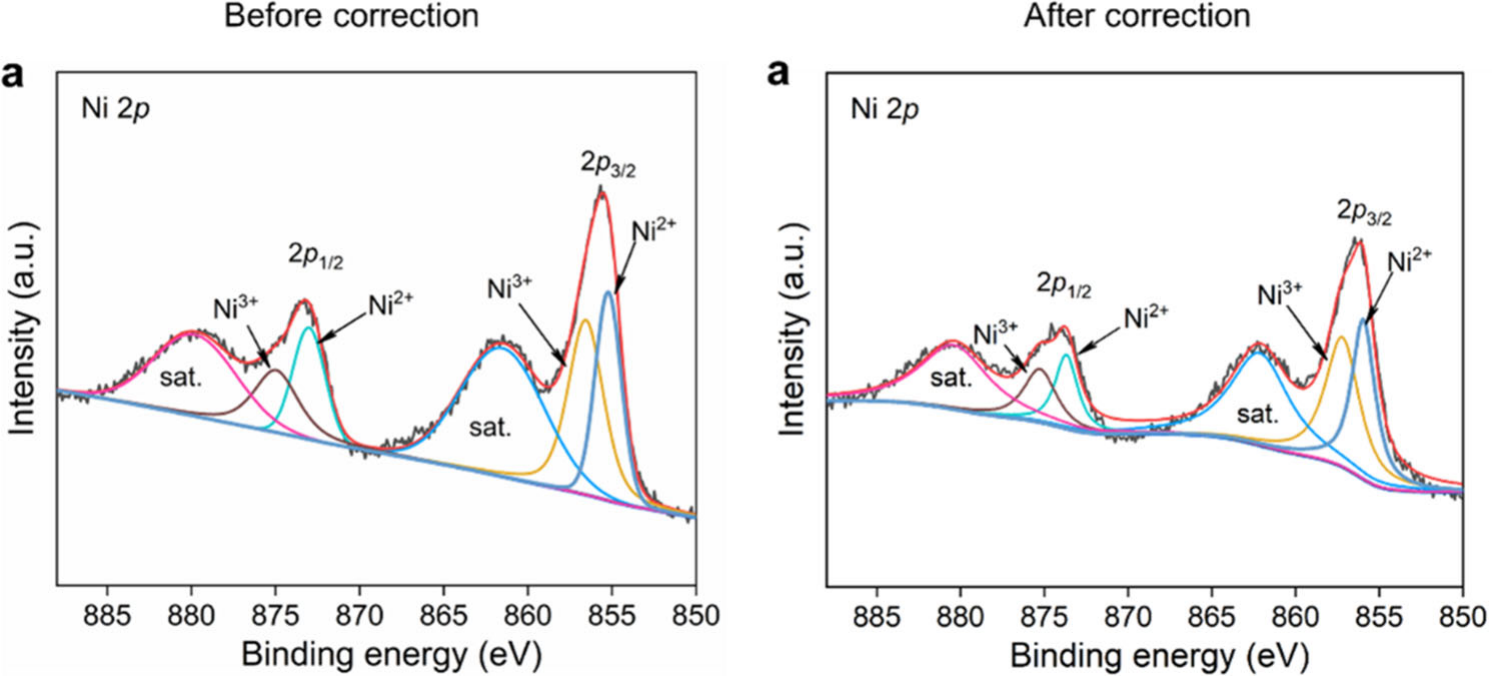


This correction does not impact the conclusions drawn in the work.

## Supplementary information

Corrected Supplementary information

## Source data

Corrected_Source_data

