# Supplementary material for: Energy-saving and product-oriented hydrogen peroxide electrosynthesis enabled by electrochemistry pairing and product engineering
Source: Nat Commun. 2023 Oct 7;14:6263. doi: 10.1038/s41467-023-41997-x (PMC10560254; doi:10.1038/s41467-023-41997-x)
Supplement: Supplementary file 1 — Supplementary Information [file 41467_2023_41997_MOESM1_ESM.pdf]

Supplementary Information for

Energy-saving and product-oriented hydrogen peroxide electrosynthesis enabled by electrochemistry pairing and product engineering

Jun Qi[1], Yadong Du[1], Qi Yang[1✉], Na Jiang[1], Jiachun Li[1], Yi Ma[1], Yangjun Ma[1], Xin Zhao[1], Jieshan Qiu[1✉]

[1]State Key Laboratory of Chemical Resource Engineering, College of Chemical Engineering, Beijing University of Chemical Technology, Beijing 100029, P. R. China.

✉ Corresponding Email: qi.yang@mail.buct.edu.cn; qiujs@mail.buct.edu.cn

Table of Contents

Economic calculation 3

Characterization of anodic catalysts 10

Performance analysis of anodic catalysts for PET upcycling 17

Characterization of cathodic catalysts 21

Performance analysis of cathodic catalysts for H_2O_2 electrosynthesis 26

Product analysis 29

Supplementary Tables 32

References 39

Economic calculation

In order to clearly prove the economic potential of the hybrid electrolysis system, we conducted a comprehensive techno-economic assessment on different electrolytic configurations. Based on past experience, we first built a relatively suitable techno-economic model to evaluate the product profitability of each system. To better approach the real industrial situation, equipment investment of electrolyzer and supporting facilities, electricity cost, electrolyte and membrane replacement cost, and maintenance cost are included in the calculation scope. In addition, separation costs that cannot be ignored are also included in the evaluation scope. For convenient considerations, the cost calculation parameters of the separation system borrowed the report that has been reported literature. On the basis of the US Department of Energy's recently announced utility-scale solar 2025 cost target[1], we estimated a cost of renewable electricity of 0.03 USD/kWh and 90% power conversion efficiency[2]. The specific calculation method is as follows.

The main operating conditions are as follows: 150 tons/day PET processing capacity, 10% impurity content, 350 days/year of operation time, 300 mA cm^{-2} current density, 50% single gas conversion efficiency, 5-year life of equipment, 35 USD/ton O_2 purchase price and 390 USD/ton waste PET purchase price[3]. Other operation parameters are as follows:

The total current. According to the processing capacity demand of PET waste and Faradaic efficiency (90%) of oxidation process. the required total current is calculated as:

$$\text{Total Current} = \frac{135000kg}{day} \times \frac{day}{86400s} \times \frac{1000g}{kg} \times \frac{mol}{192g} \times 6e^{-} \times \frac{96485C}{mol} \times \frac{1}{0.9} = 5234646.3\,A \qquad (1)$$

O_2 demand.

$$O_2 \text{ flow rate} = 5234646.3A \times \frac{1}{2e^{-}} \times \frac{mol}{96485\,C} \times \frac{0.032\,kg}{mol} \times \frac{86400\,s}{day} = 75000.0\,kg/day \qquad (2)$$

Outlet liquid product flow rate. According to the performance of Ni_1Mn_1-MOF-Se/NF catalyst, the average Faradaic efficiency of formate from EG is set to 90%, and the yields of KDF and PTA are 70% and 95%, respectively.

$$A\text{node KDF flow rate} = 5234646.3A \times 0.9 \times 0.7 \times \frac{1}{6e^{-}} \times \frac{mol}{96485C} \times \frac{0.130kg}{mol} \times \frac{3600s}{hr} = 2666.0\,kg/hr \qquad (3)$$

$$\text{PTA flow rate} = \frac{150000\text{kg}}{day} \times \frac{mol}{0.192kg} \times 0.9 \times 0.95 \times \frac{0.166kg}{mol} \times \frac{day}{24hr} = 4620.2\,kg/hr \qquad (4)$$

$$\text{Formic acid flow rate} = 5234646.3A \times 0.9 \times \frac{1}{6e^{-}} \times \frac{mol}{96485\,C} \times \frac{0.046\,kg}{mol} \times \frac{3600\,s}{hr} = 1347.7\ kg/hr \quad (5)$$

$$\text{Cathode } H_2O_2 \text{ flow rate} = 5234646.3A \times 0.93 \times \frac{1}{2e^{-}} \times \frac{mol}{96485C} \times \frac{0.034kg}{mol} \times \frac{3600s}{hr} = 3087.9\ kg/hr \quad (6)$$

Electrolyzer area.

$$\text{Electrolyzer area} = 5234646.3A \times \frac{1}{0.3A/cm^2} \times \frac{m^2}{10000cm^2} = 1744.9\ m^2 \quad (7)$$

Raw material cost.

$$O_2 \text{ cost} = 75000.0\ \frac{kg}{day} \times 350\,day \times \frac{0.035\ USD}{kg} = 918750.0\ USD \quad (8)$$

$$\text{PET cost} = 150000\ \frac{kg}{day} \times 350\,day \times \frac{0.39\ USD}{kg} = 20475000\ USD \quad (9)$$

$$\text{Formic acid cost} = 32344.8\ \frac{kg}{day} \times 350\,day \times \frac{0.74\ USD}{kg} = 8377303\ USD \quad (10)$$

Capital cost. Since both anode and cathode catalysts are non-precious metal-based materials, and their cost is negligible compared with that of noble metal Pt and Ir. Here, we use 460 USD/kW as the stack cost[4]. The reference electrolyzer operates at 1.75 V and 0.4 A cm^{-2} and the installation factor is 1.12. The balance of plant (BoP) cost is assumed to be 35% of the total cost of the electrolyzer system, and these values were derived from the H_2A model. PSA cost is related to the outlet gas flow rate, estimated according to the literature as 1989043 USD/(1000 m^3/hr)[4]. Considering that the generated formic acid reacts with KOH to form formate, it is necessary to adjust the pH with formic acid first to precipitate PTA, and the supernatant is further sent to a vacuum distillation device for separation to obtain KDF. We assume that the capital cost of the separation process accounts for 20% of the raw material cost[5].

1. Electrolyzer cost

$$\text{Electrolyzer cost} = 460\frac{USD}{kW} \times \frac{0.4\text{A}}{cm^2} \times 1.75 \times \frac{10^4 cm^2}{m^2} \times \frac{kW}{1000W} \times 1308.7m^2 = 4214014\ USD \quad (11)$$

2. Balance of plant

$$\text{Balance of plant cost} = 4214014 \times \frac{0.35}{0.65} = 2269084.5\ USD \quad (12)$$

3. Cost of liquid product separation. Under conventional circumstances, the complexity and high energy consumption limit the separation of hydrogen peroxide from alkaline electrolyte to a certain extent. However, the electrolyte containing H_2O_2 proposed in this study

can be directly used for the downstream production of sodium peroxyborate and dibenzoyl peroxide, which avoids the separation problem. The converting from H_2O_2 to SPB and BPO can achieve profit growth. To simplify the economic evaluation and conduct parallel comparisons, we exclude both the raw material cost (borax, benzoyl chloride) and the product values (SPB, BPO). For the separation of anode products, the corresponding equipment cost can be assumed to be 20% of the raw material cost.

$$\text{Cost of liquid separation} = 20475\,000\,USD \times 0.2 = 4095\,000\ USD \tag{13}$$

Operating cost. The working and maintenance cost was assumed to be 2.5% of the capital cost. According to the literature, membrane cost can be equivalent to 180 USD/m^2 when reference operating conditions are considered. As mentioned above, the separation of cathode products need not be considered, but the purification of anode PTA and KDF is an energy intensive separation process. Thus, we assume the utilities cost account for 60% of the raw material cost[5]. Given the loss of electrolyte due to neutralization, it is assumed that the consumption cost of electrolyte and water accounts for 30% of the cost of raw materials.

1. Electricity

$$\begin{aligned}\text{Electricity cost} &= 0.861V \times \frac{0.3A}{cm^2} \times 1744.9m^2 \times \frac{10000cm^2}{m^2} \times \frac{kW}{1000W} \times 24 \times 350 \\ &\times 0.03\frac{USD}{kW\ h} \times \frac{1}{0.9} = 1261981.5\,USD\end{aligned} \tag{14}$$

2. Working and maintenance cost

$$\text{Cost} = 6483\,098.5USD \times 0.025 = 162077.5\,USD \tag{15}$$

3. Cost of liquid separation

$$\text{Cost} = 20475000\ USD \times 0.6 = 12285\,000\ USD \tag{16}$$

4. Water and electrolyte cost

$$\text{Cost} = 20475000\ USD \times 0.3 = 6142500\ USD \tag{17}$$

5. Membrane replacement

$$\text{Cost} = 180\frac{USD}{\text{m}^2} \times 1744.9m^2 = 314082\,USD \tag{18}$$

Other cost. Other cost, such as operating overhead, labor-related cost and selling (or transfer) expense, account for 20% of the cost of raw materials.

$$\text{Cost} = (918750.0\,USD + 20475000\,USD) \times 0.2 = 4278750.0\,USD \tag{19}$$

Product sales. Based on market prices and literature references, the selling prices of KDF, PTA, and H_2O_2 are set at 1590 USD/ton, 1260 USD/ton,[3] and 1200 USD/ton[6, 7].

$$\text{KDF sales} = 1590\,\frac{USD}{ton} \times \frac{ton}{1000\,kg} \times (2666.0)\,\frac{kg}{hr} \times 24\,hr \times 350\,day = 35607096\,USD \tag{20}$$

$$\text{PTA sales} = 1260\,\frac{USD}{ton} \times \frac{ton}{1000\,kg} \times 4620.2\,\frac{kg}{hr} \times 24\,hr \times 350\,day = 48900196.8\,USD \tag{21}$$

$$H_2O_2\ \text{sales} = 1200\,\frac{USD}{ton} \times \frac{ton}{1000\,kg} \times 3087.9\,\frac{kg}{hr} \times 24\,hr \times 350\,day = 31126032\,USD \tag{22}$$

ORR to H_2O_2 coupled with OER. For cross-sectional comparison, the PET throughput and operating conditions of the plant are consistent with the above strategy. Other operation parameters and economic data are as follows:

O_2 flow rate at anode side. Faraday efficiency of the anode product O_2 can be assumed to be 100%.

$$O_2\ \text{flow rate} = 5234646.3\,A \times \frac{1}{4e^-} \times \frac{mol}{96485\,C} \times \frac{0.032\,kg}{mol} \times \frac{86400\,s}{day} = 37500.0\ kg/day \tag{23}$$

Outlet H_2O_2 flow rate.

$$H_2O_2\ \text{flow rate} = 5234646.3\,A \times 0.93 \times \frac{1}{2e^-} \times \frac{mol}{96485\,C} \times \frac{0.034\,kg}{mol} \times \frac{3600\,s}{hr} = 3087.9\ kg/hr \tag{24}$$

Capital cost. Similar to the ORR//PET oxidation system, 460 USD/kW can be used as the stack cost. In contrast, the anode products are all gas phase, so no liquid separation device can be ignored.

1. Electrolyzer cost

$$\text{Electrolyzer cost} = 460\,\frac{USD}{kW} \times \frac{0.4\text{A}}{cm^2} \times 1.75 \times \frac{10^4\,cm^2}{m^2} \times \frac{kW}{1000\,W} \times 1308.7\,m^2 = 4214014\,USD \tag{25}$$

2. Balance of plant

$$\text{Balance of plant cost} = 4214014 \times \frac{0.35}{0.65} = 2269084.5\,USD \tag{26}$$

Operating cost.

1. Electricity

$$\text{Electricity cost} = 1.056\,V \times \frac{0.3\,A \times 10000}{m^2} \times 1744.9\,m^2 \times \frac{kW}{1000\,W} \times 24 \times 350 \times 0.03\,\frac{USD}{kW\ h} \times \frac{1}{0.9} = 1547796.1\,USD \tag{27}$$

2. Working and maintenance cost

$$\text{Cost} = 16647783\ USD \times 0.025 = 162077.5\ USD \tag{28}$$

3. Water and electrolyte cost

$$\text{Cost} = 918750\ USD \times 0.3 = 275625\ USD \tag{29}$$

4. Membrane replacement

$$\text{Cost} = 180\frac{USD}{\text{m}^2} \times 1744.9 m^2 = 314082\ USD \tag{30}$$

Other Cost. Other cost, such as operating overhead, labor-related cost and selling (or transfer) expense, account for 20% of the cost of raw materials.

$$\text{Cost} = 918750 USD \times 0.2 = 183750\ USD \tag{31}$$

Product Sales. Based on current market prices and literature references, the selling price of O_2 is set at 35 USD/ton.

$$\text{H}_2\text{O}_2 \text{ sales} = 1200\ \frac{USD}{ton} \times \frac{ton}{1000\ kg} \times 3087.9 \frac{kg}{hr} \times 24\,hr \times 350\,day = 31126032\ USD \tag{32}$$

$$\text{O}_2 \text{ sales} = 35\ \frac{USD}{ton} \times \frac{ton}{1000\ kg} \times 37500\ \frac{kg}{day} \times 350\,day = 459375\ USD \tag{33}$$

Green hydrogen production coupled with PET upcycling. Many data including processing capacity and equipment parameters are consistent with the above system. Assuming that the Faradaic efficiency of the cathode H_2 is 100%, the market price of hydrogen is 1900 USD/ton.

$$\mathrm{H_2\ flow\ rate} = 5234646.3A \times \frac{1}{2e^-} \times \frac{mol}{96485\,C} \times \frac{0.002\,kg}{mol} \times \frac{86400\,s}{day} = 4687.5\ kg/day \tag{34}$$

Raw material cost.

$$\mathrm{PET\ cost} = 150000\,\frac{kg}{day} \times 350\,day \times \frac{0.39\,USD}{kg} = 20475000\ USD \tag{35}$$

Capital cost. Under ideal conditions, hydrogen as the only product of the cathode does not need to be separated by pressure swing adsorption, so only the liquid phase product needs to be separated.

1. Electrolyzer cost

$$\mathrm{Electrolyzer\ cost} = 460\,\frac{USD}{kW} \times \frac{0.4\mathrm{A}}{cm^2} \times 1.75 \times \frac{10^4\,cm^2}{m^2} \times \frac{kW}{1000\,W} \times 1308.7m^2 = 4214014\ USD \tag{36}$$

2. Balance of plant

$$\mathrm{Balance\ of\ plant\ cost} = 4214014 \times \frac{0.35}{0.65} = 2269084.5\,USD \tag{37}$$

3. Cost of liquid product separation (distillation column, decanter and its supporting equipment)

$$\mathrm{Cost\ of\ liquid\ separation} = 20475000USD \times 0.2 = 4095000\,USD \tag{38}$$

Operating cost.

1. Electricity

$$\mathrm{Electricity\ cost} = 1.768V \times \frac{0.3A}{cm^2} \times 1744.9m^2 \times \frac{10000\,cm^2}{m^2} \times \frac{kW}{1000W} \times 24 \times 350 \times 0.03\,\frac{USD}{kW\ h} \times \frac{1}{0.9} = 2591385.9\,USD \tag{39}$$

2. Working and maintenance cost

$$Cost = 10578098.5\,USD \times 0.025 = 264452.5\,USD \tag{40}$$

3. Cost of liquid separation

$$Cost = 20475000\ USD \times 0.6 = 12285000\ USD \tag{41}$$

4. Water and electrolyte cost

$$Cost = 20475000\ USD \times 0.3 = 6142500\ USD \tag{42}$$

Other cost. Since the HER//EOR system is simpler in process and equipment than the ORR//PET oxidation system, we assume that other costs account for 10% of the cost of raw materials.

$$Cost = 20475000\ USD \times 0.1 = 2047500\ USD \tag{43}$$

Product Sales.

$$\text{KDF sales} = 1590\ \frac{USD}{ton} \times \frac{ton}{1000\ kg} \times (2666.0)\frac{kg}{hr} \times 24\,hr \times 350\,day = 35607096\ USD \tag{44}$$

$$\text{PTA sales} = 1260\ \frac{USD}{ton} \times \frac{ton}{1000\ kg} \times 4620.2\frac{kg}{hr} \times 24\,hr \times 350\,day = 48900196.8\ USD \tag{45}$$

$$H_2\ sales = 1900\ \frac{USD}{ton} \times \frac{ton}{1000\ kg} \times 4687.5\frac{kg}{day} \times 350\,day = 3117187.5\ USD \tag{46}$$

Characterization of anodic catalysts

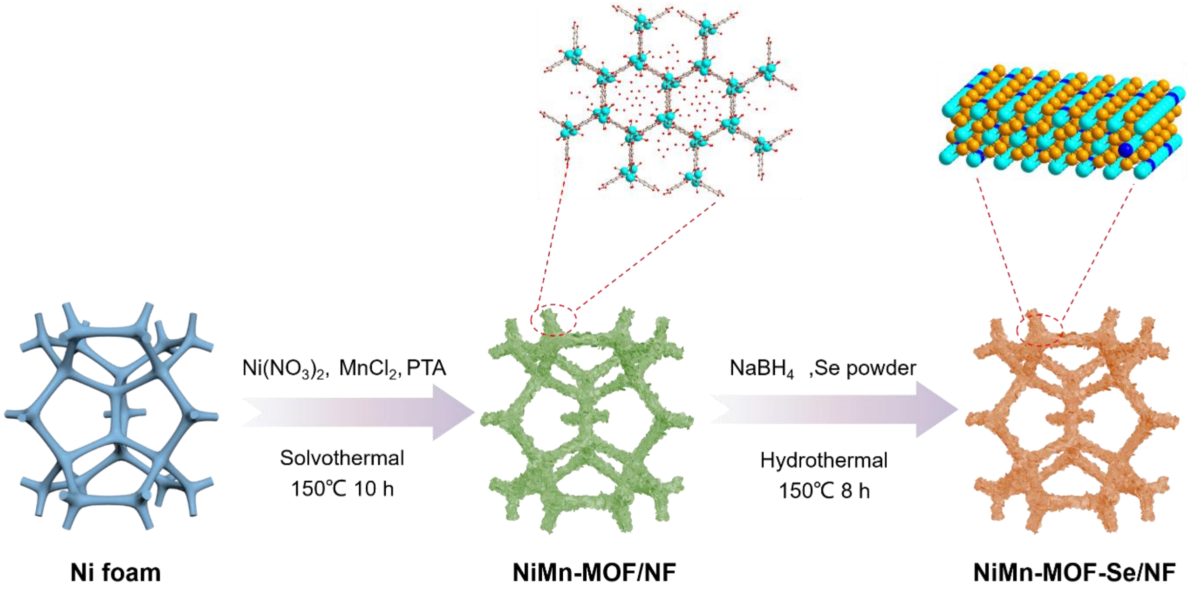

Supplementary Fig. 1 Schematic illustration of the preparation of the NiMn-MOF-Se/NF catalyst. The detailed material synthesis steps can be seen from the Methods. Considering the poor water solubility of PTA, 15 mL DMF, 5 mL ethanol, and 10 mL deionized water were selected as the mixed solvent for solvothermal synthesis in the first step.

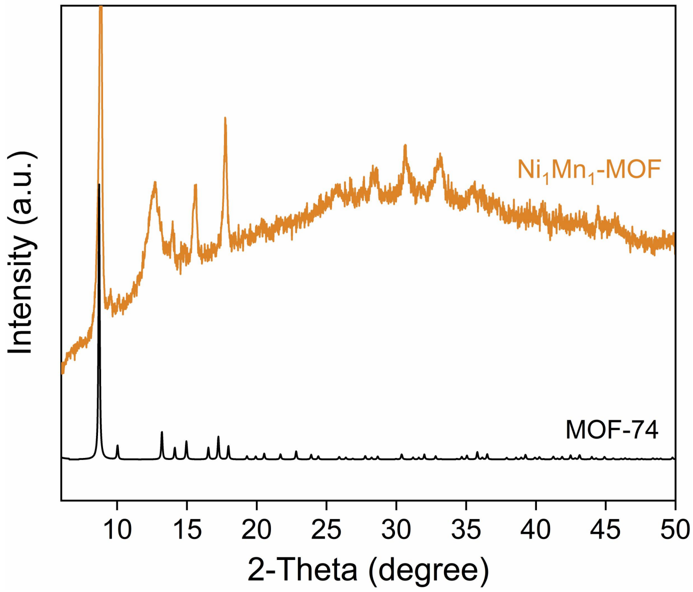

Supplementary Fig. 2 XRD patterns of the Ni$_1$Mn$_1$-MOF catalyst and the standard Ni-MOF-74.

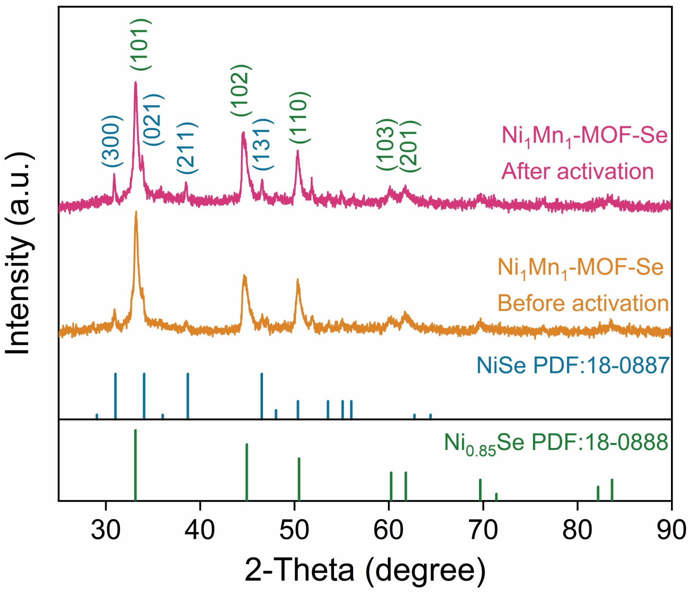

Supplementary Fig. 3 XRD patterns of the Ni_1Mn_1-MOF-Se catalysts before and after activation.

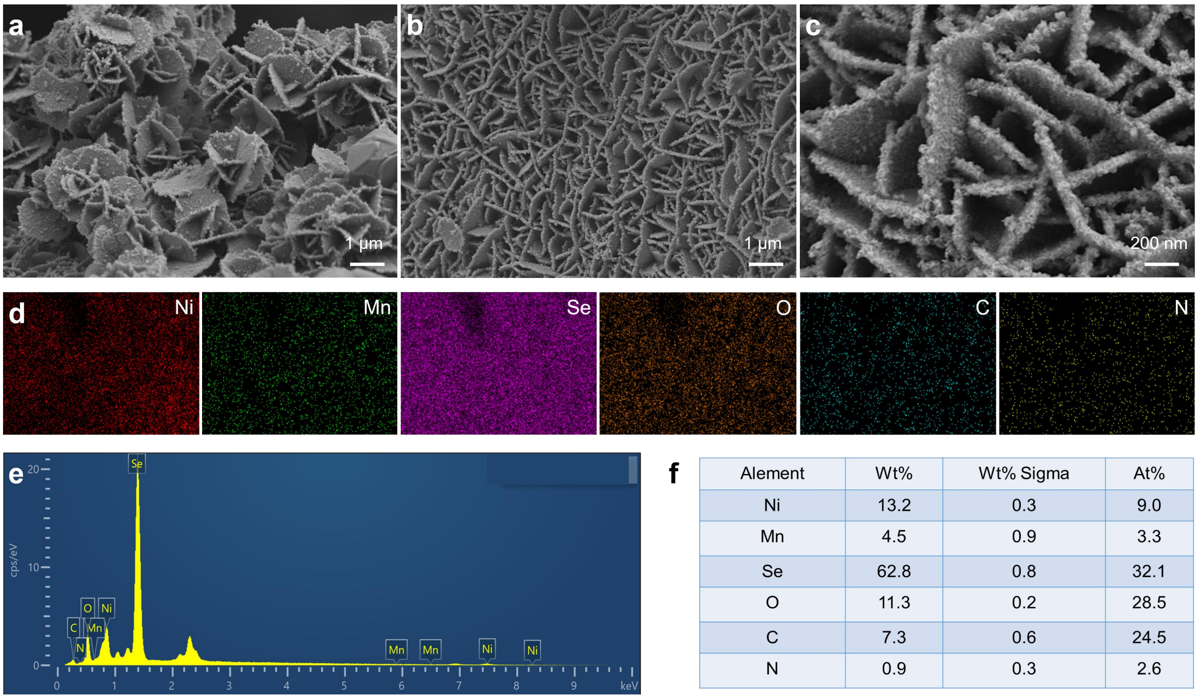

Alement	Wt%	Wt% Sigma	At%
Ni	13.2	0.3	9.0
Mn	4.5	0.9	3.3
Se	62.8	0.8	32.1
O	11.3	0.2	28.5
C	7.3	0.6	24.5
N	0.9	0.3	2.6

Supplementary Fig. 4 Morphology characterizations of Ni_1Mn_1-MOF-Se/NF. SEM images (**a-c**) and the corresponding elemental mappings (**d-f**) of the Ni_1Mn_1-MOF-Se/NF catalyst.

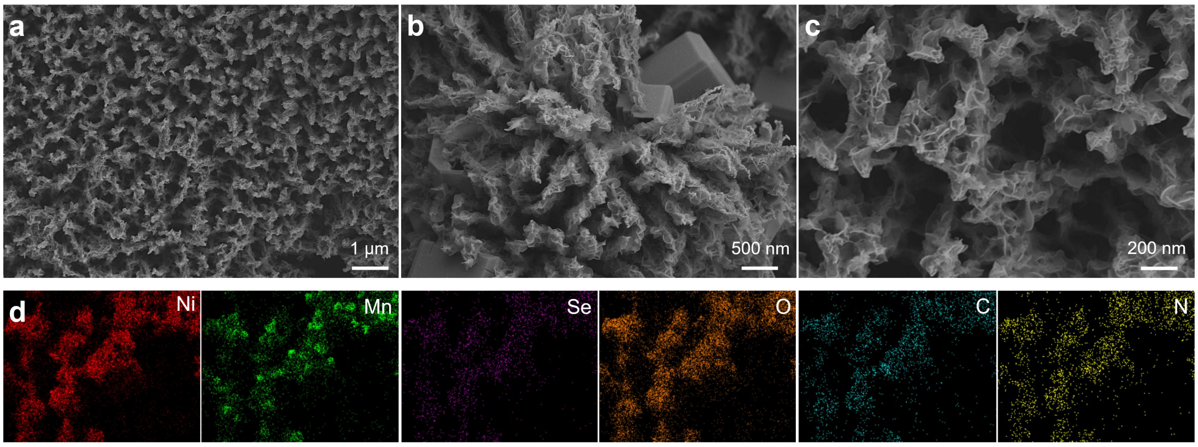

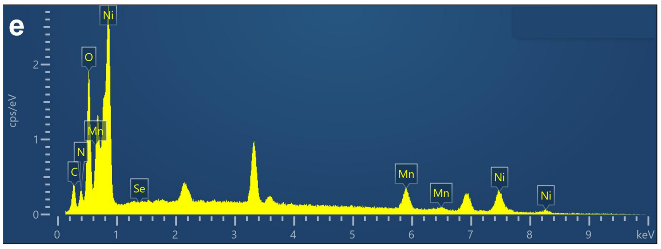

f

Alement	Wt%	Wt% Sigma	At%
Ni	41.3	0.9	20.7
Mn	28.0	1.3	15.0
Se	0.0	0.1	0.0
O	15.4	0.5	28.3
C	10.5	1.5	25.8
N	4.8	0.5	10.2

Supplementary Fig. 5 Morphology characterizations of Ni_1Mn_1-MOF-Se/NF after activation. SEM images (**a-c**) and the corresponding elemental mappings (**d-f**) of the Ni_1Mn_1-MOF-Se/NF catalyst after activation. The results of element mappings show that the atomic content of Se is 0 at%, while a small amount of Se element can still be seen in the Supplementary Fig. 5d. Therefore, XPS and ICP-OES are needed to continue determining changes in surface Se content.

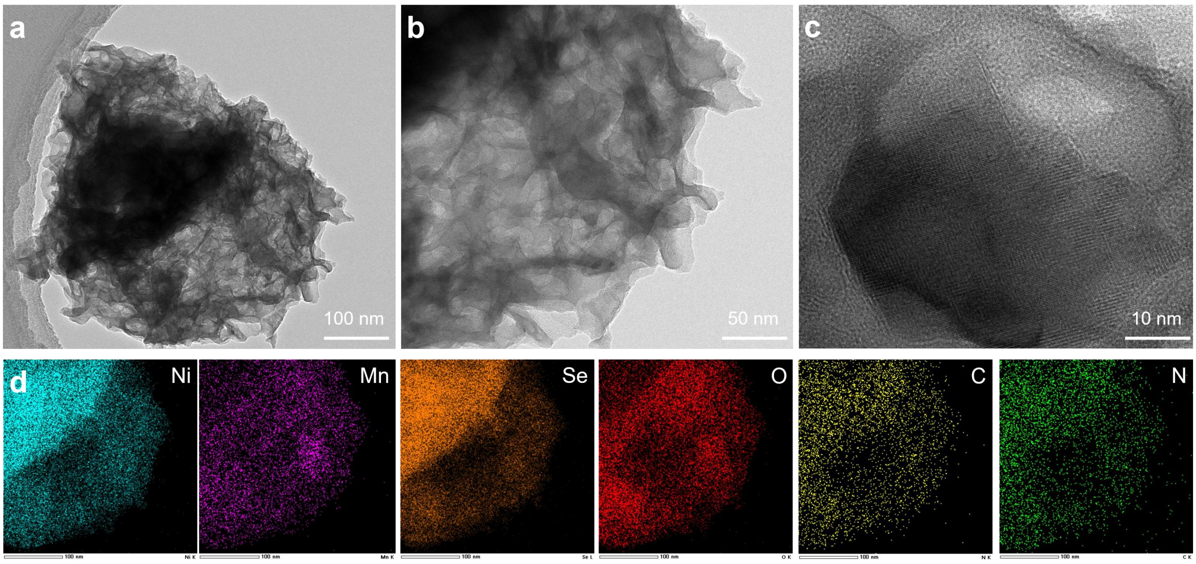

Supplementary Fig. 6 Microstructure characterization of Ni_1Mn_1-MOF-Se/NF before activation. a, b TEM images. **c** HR-TEM image. **d** EDS mappings.

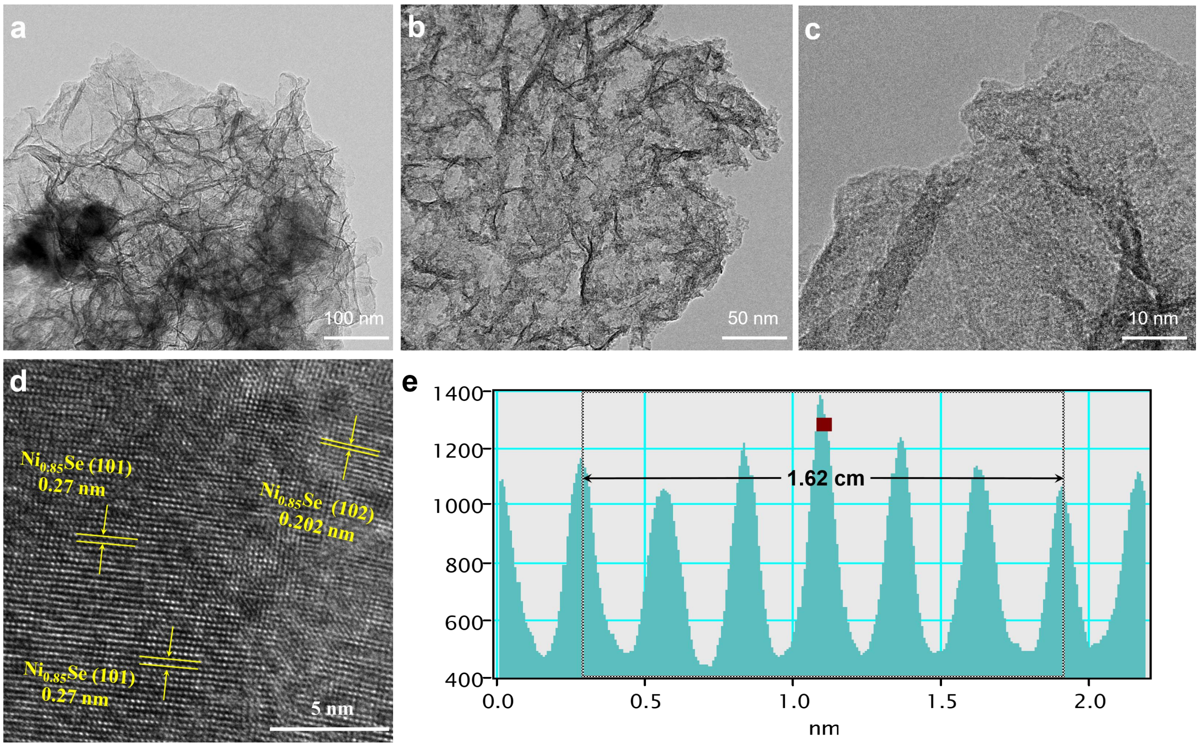

Supplementary Fig. 7 Microstructure of Ni_1Mn_1-MOF-Se/NF after activation. a, b TEM images. **c, d** HR-TEM images. **e** Measurement of lattice spacing.

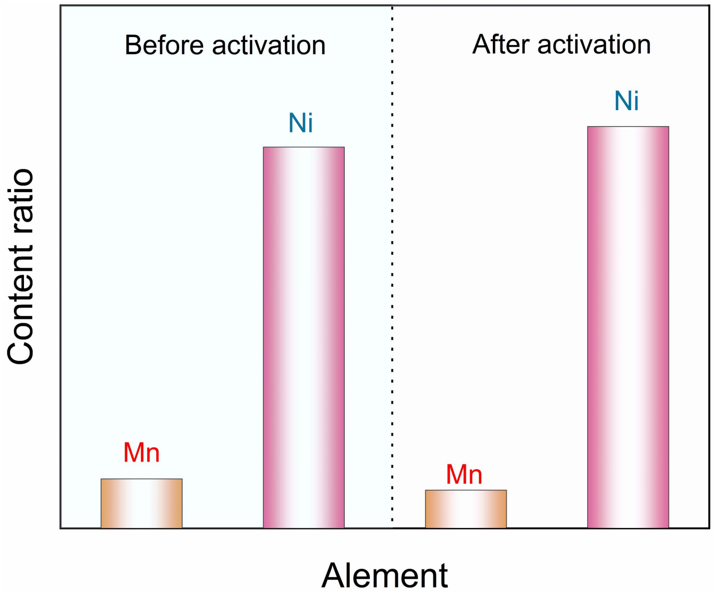

Supplementary Fig. 8 Elemental analysis of Ni_1Mn_1-MOF-Se/NF before and after activation. ICP-OES results of the Ni_1Mn_1-MOF-Se/NF catalyst before and after activation.

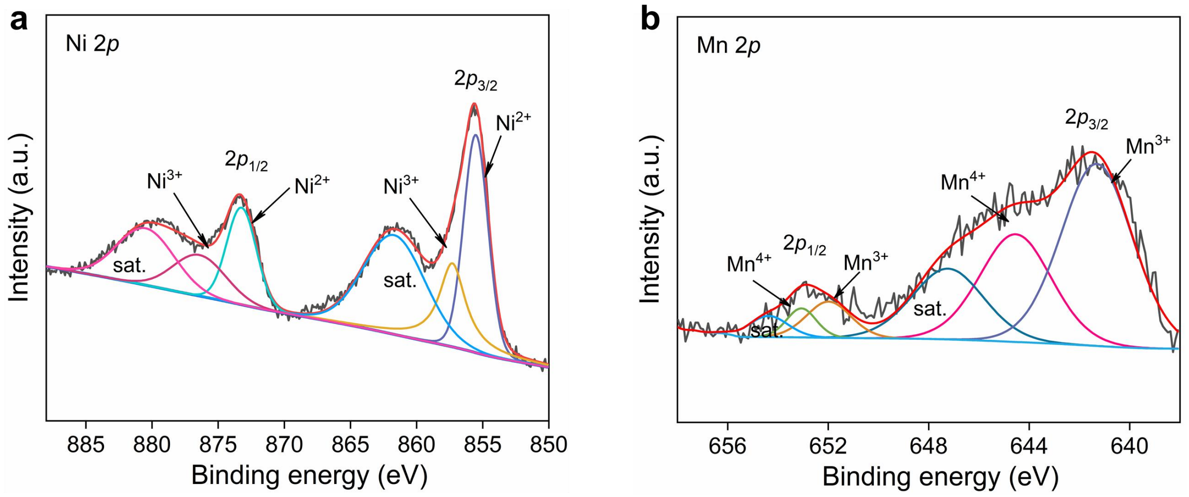

Supplementary Fig. 9 Characterizations of Ni and Mn valent state of Ni_1Mn_1-MOF/NF. Ni 2*p* (**a**) and Mn 2*p* (**b**) XPS spectra of the Ni_1Mn_1-MOF/NF catalyst.

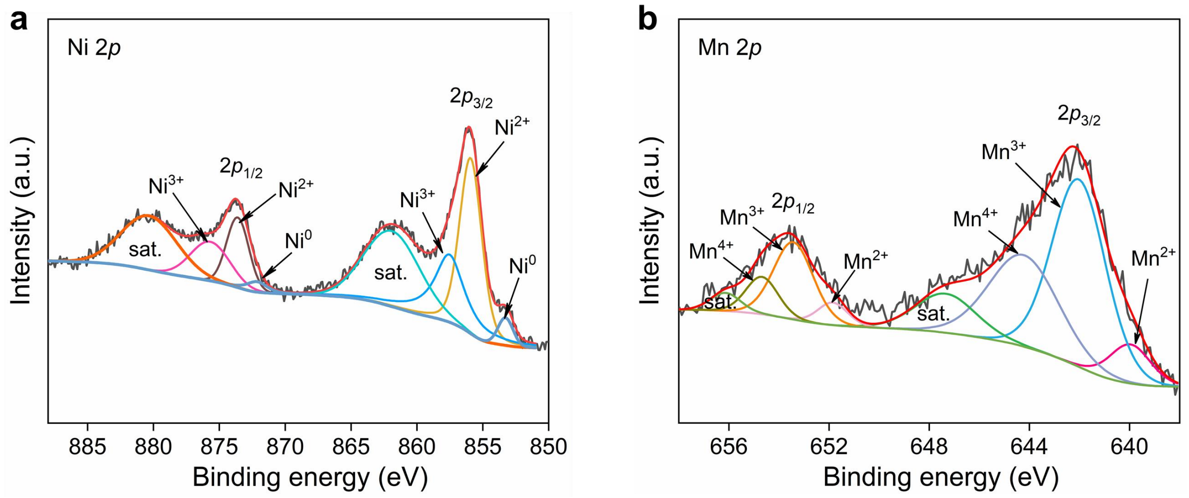

Supplementary Fig. 10 Characterizations of Ni and Mn valent state of Ni_1Mn_1-MOF-Se/NF. Ni 2*p* (**a**) and Mn 2*p* (**b**) XPS spectra of the Ni_1Mn_1-MOF-Se/NF catalyst.

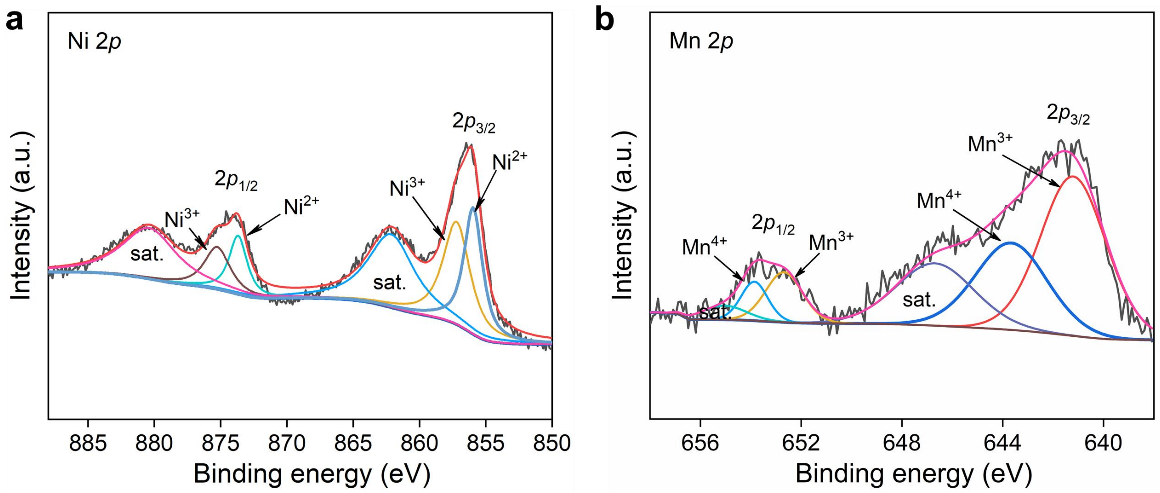

Supplementary Fig. 11 Characterizations of Ni and Mn valent state of Ni_1Mn_1-MOF-Se/NF after activation. Ni 2*p* (**a**) and Mn 2*p* (**b**) XPS spectra of the Ni_1Mn_1-MOF-Se/NF catalyst after activation. Compared with the Ni_1Mn_1-MOF-Se/NF before activation, Ni^0 evolves into Ni^{2+} and Ni^{3+} after the electrochemical activation, representing the increased content of NiOOH as the electrochemically active species. Similarly, the valence state of Mn has also been significantly improved[8, 9].

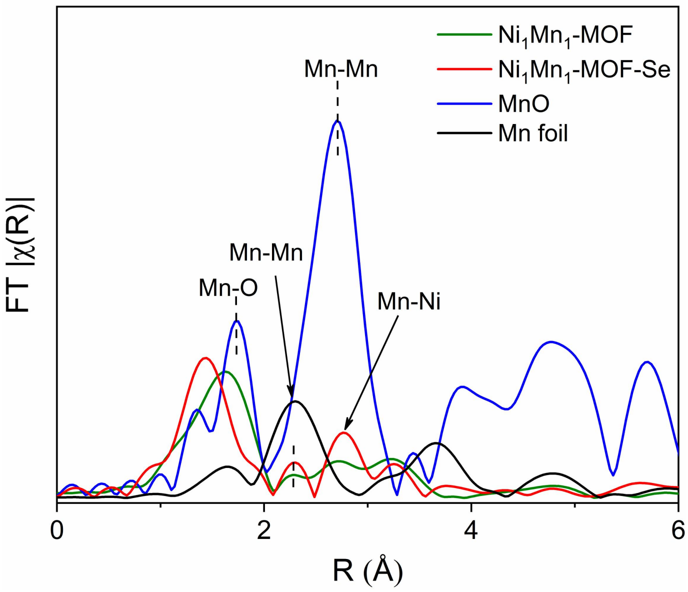

Supplementary Fig. 12 XAS characterizations of Ni_1Mn_1-MOF/NF and Ni_1Mn_1-MOF-Se/NF. Mn K-edge EXAFS spectra of Ni_1Mn_1-MOF and Ni_1Mn_1-MOF-Se.

Performance analysis of anodic catalysts for PET upcycling

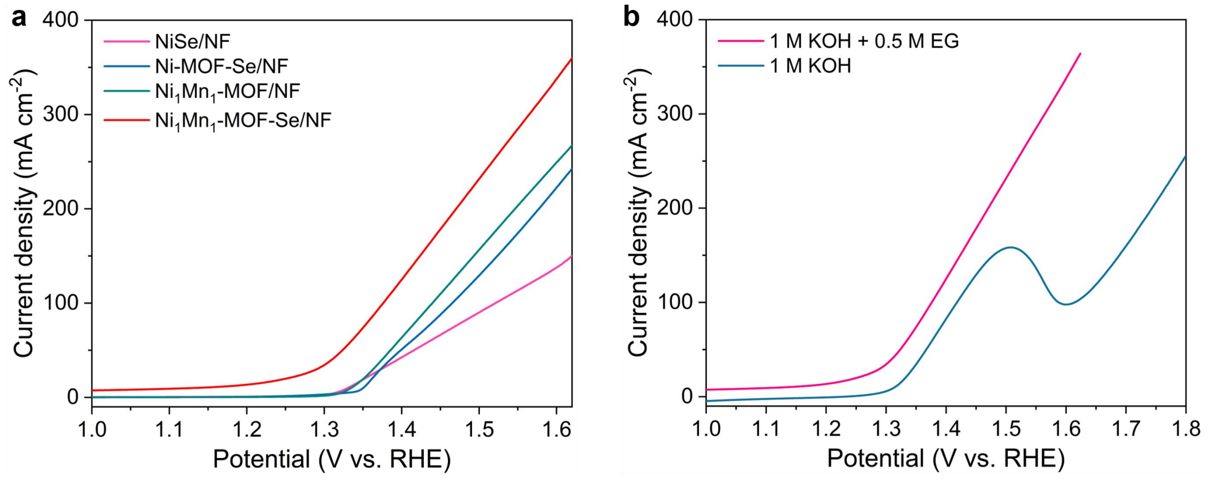

Supplementary Fig. 13 EG oxidation performance of different catalysts. a Polarization curves of different catalysts for EG oxidation without *iR* compensation. **b** Polarization curves of the Ni_1Mn_1-MOF-Se/NF catalyst for EG oxidation and OER without *iR* compensation.

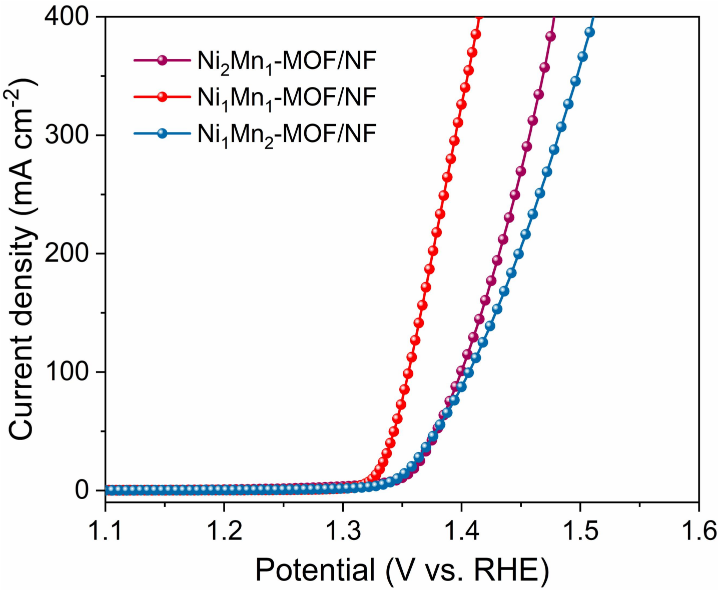

Supplementary Fig. 14 EOR performance of catalysts with different Ni/Mn ratios. Polarization curves of NiMn-MOF/NF with different Ni and Mn ratios for EG oxidation.

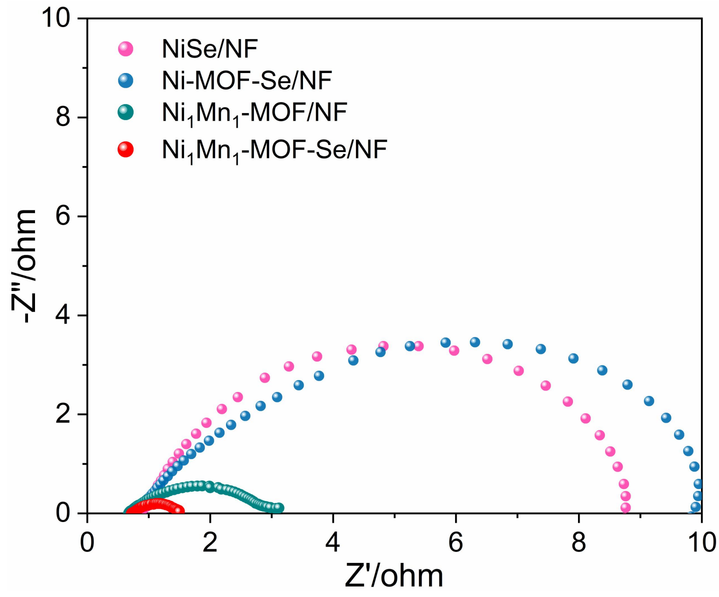

Supplementary Fig. 15 Electrochemical impendence spectra (EIS) measurement of different catalysts for studying the electron transfer capability. Nyquist plots of different catalysts for EG oxidation under 1.3 V (vs. RHE).

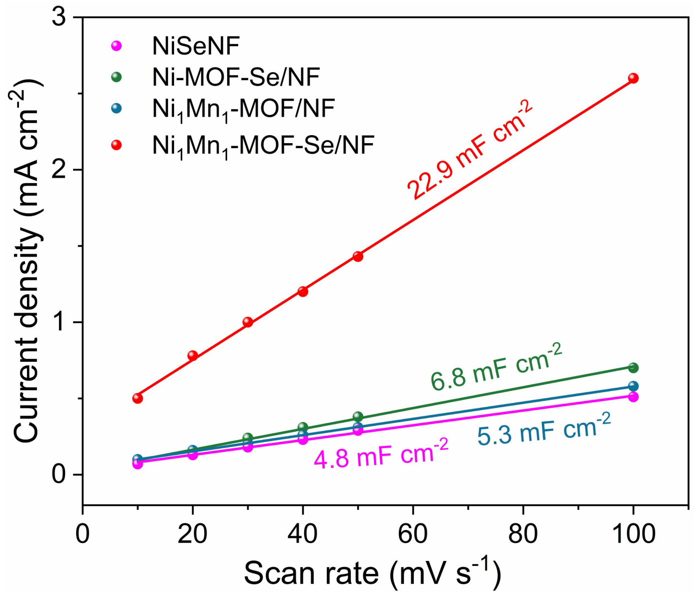

Supplementary Fig. 16 Determination of double layer capacitance as an indicator of the electrochemically active area of catalysts. Double-layer charging current plotted against the CV scan rate for different catalysts.

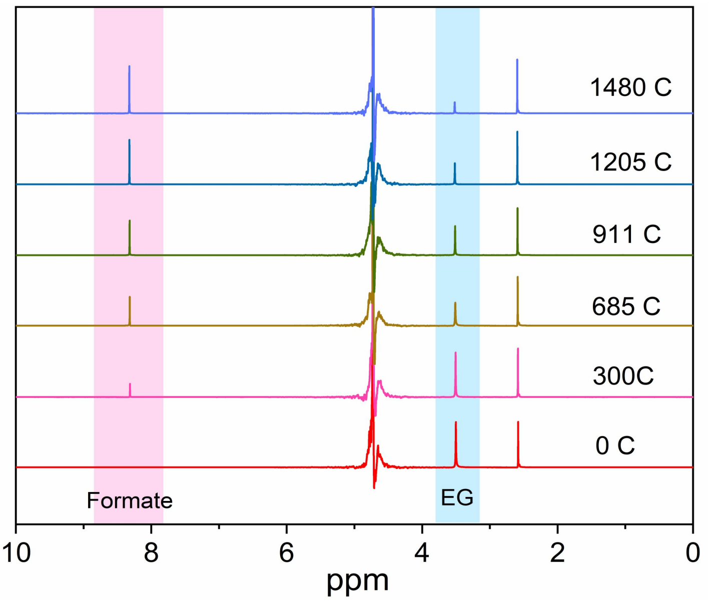

Supplementary Fig. 17 Product analysis of EG oxidation. ^{1}H NMR measurements of EG oxidation with Ni_1Mn_1-MOF-Se/NF as catalyst at 1.4 V (vs. RHE). Detailed sample preparation and detection steps can refer to Product analysis, in which DMSO is selected as the internal standard, and the corresponding peak location is around 2.5 ppm. The peak at 3.5 ppm can be signed in EG, and the peak at 8.3 ppm can be signed in product formate.

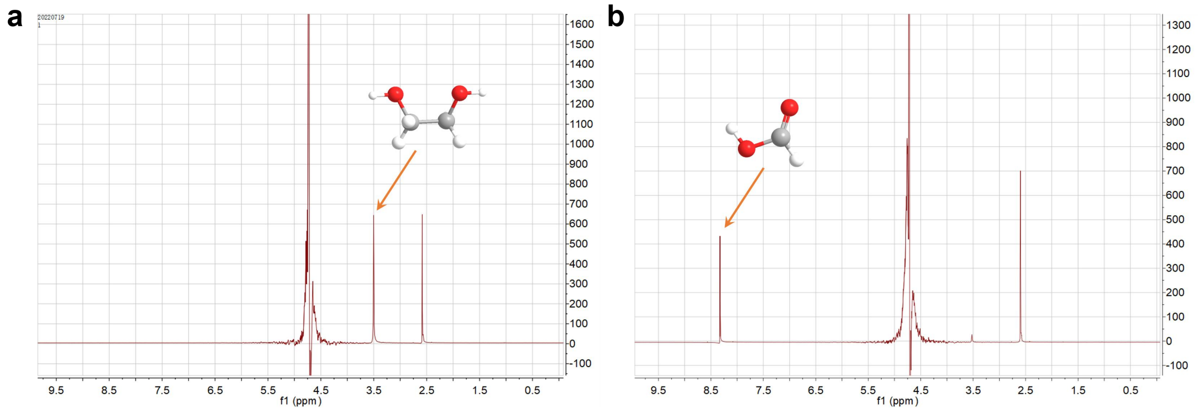

Supplementary Fig. 18 Product analysis results. ^{1}H NMR measurements of EG oxidation with Ni_1Mn_1-MOF-Se/NF before (**a**) and after oxidation (**b**).

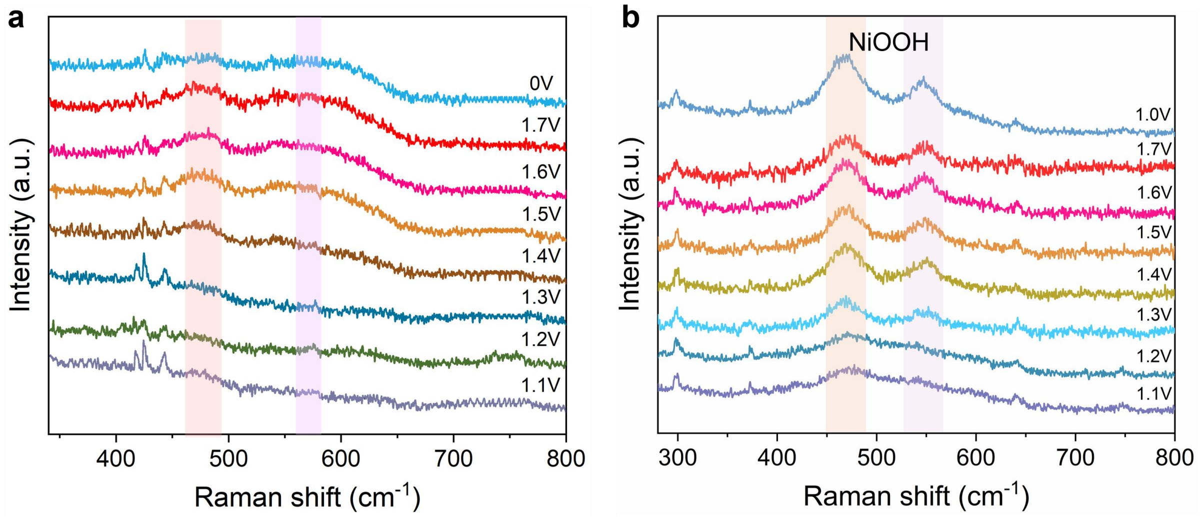

Supplementary Fig. 19 In situ characterization uncovering the evolution of the catalyst. In situ Raman spectra of the Ni_1Mn_1-MOF-Se/NF catalyst in 1 M KOH with 0.5 M EG electrolyte for EG oxidation (**a**) and 1 M KOH for OER (**b**).

Characterization of cathodic catalysts

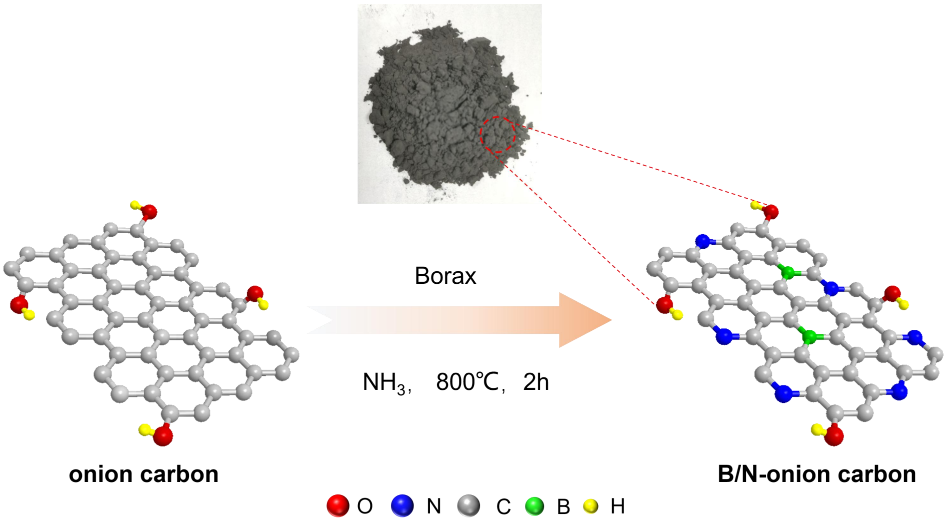

Supplementary Fig. 20 Schematic diagram of the B/N-onion carbon catalyst preparation. The detailed material synthesis steps can be seen from the Methods. For the purchased commercial onion carbon, first oxygen plasma treatment for 30 minutes was completed to increase the oxygen-containing functional groups and hydrophilicity of its surface. The treated onion carbon was mixed with borax in a ratio of 1:5 through grinding and then placed in a tubular furnace for thermal annealing under NH_3 atmosphere.

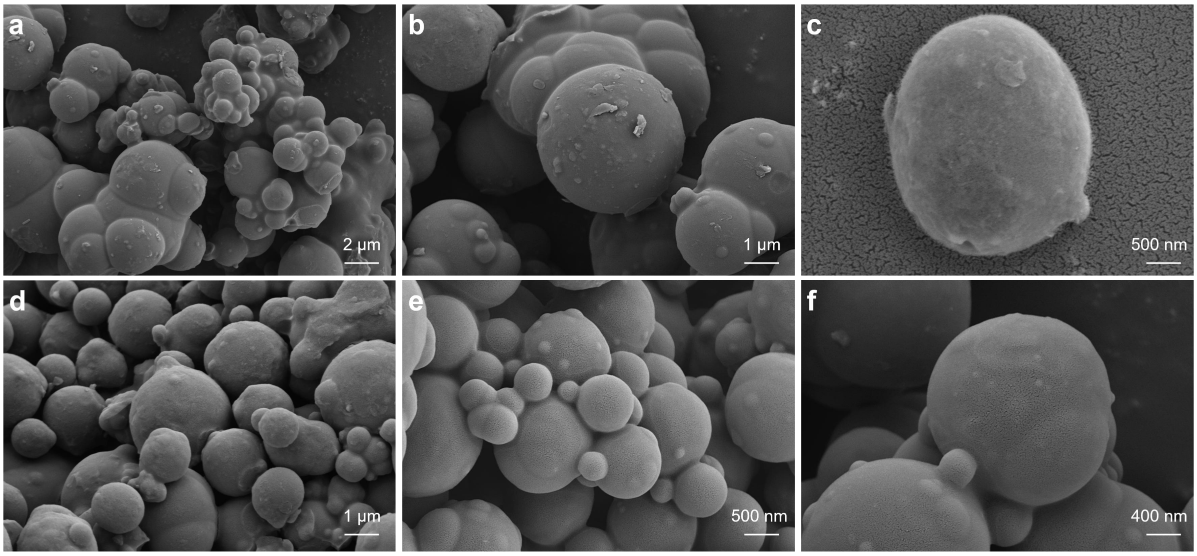

Supplementary Fig. 21 Characterizations of different catalysts. SEM images of onion

carbon (**a-c**) and N-onion carbon catalyst (**d-f**).

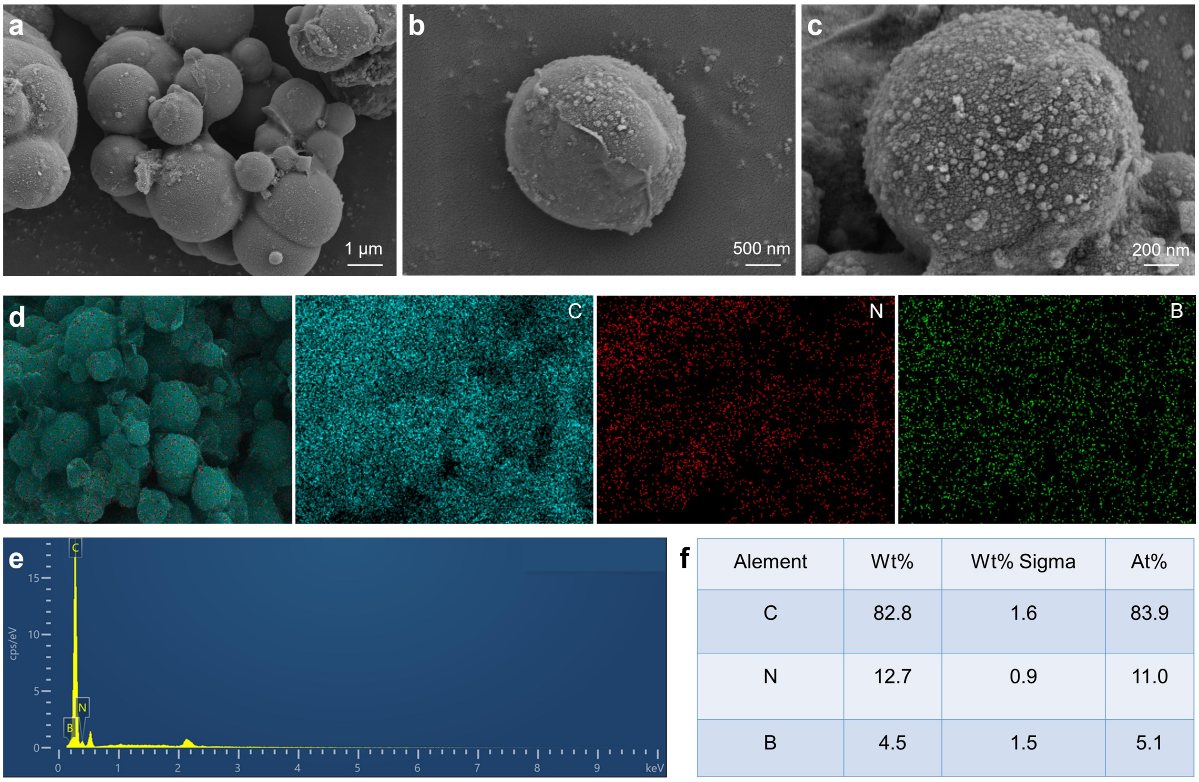

Alement	Wt%	Wt% Sigma	At%
C	82.8	1.6	83.9
N	12.7	0.9	11.0
B	4.5	1.5	5.1

Supplementary Fig. 22 Characterizations of B/N-onion carbon. SEM images (**a-c**) and the corresponding elemental mappings (**d-f**) of the B/N-onion carbon catalyst.

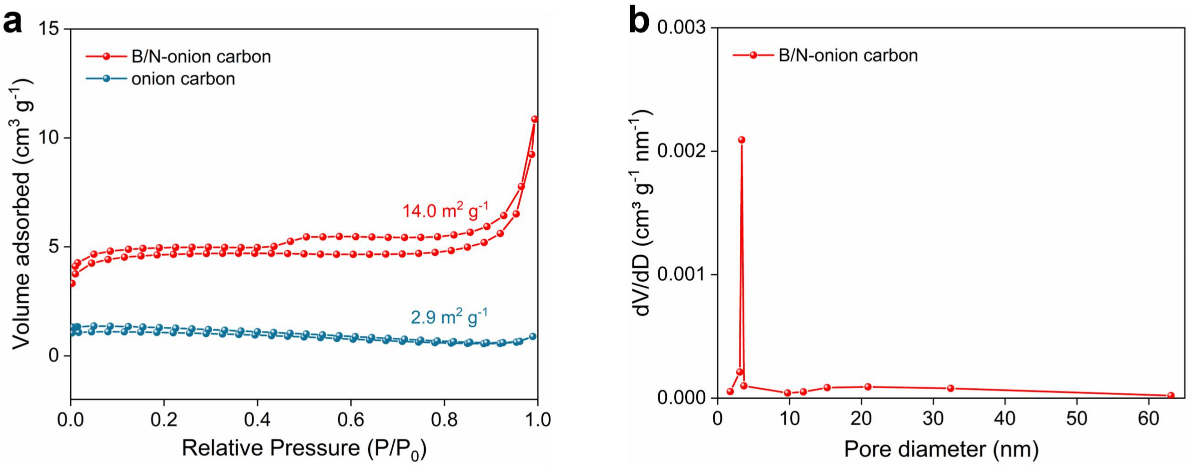

Supplementary Fig. 23 Characterization of onion carbon and B/N-onion carbon. a Nitrogen adsorption-desorption isotherms of onion carbon and B/N-onion carbon. **b** Pore

distribution pattern of B/N-onion carbon.

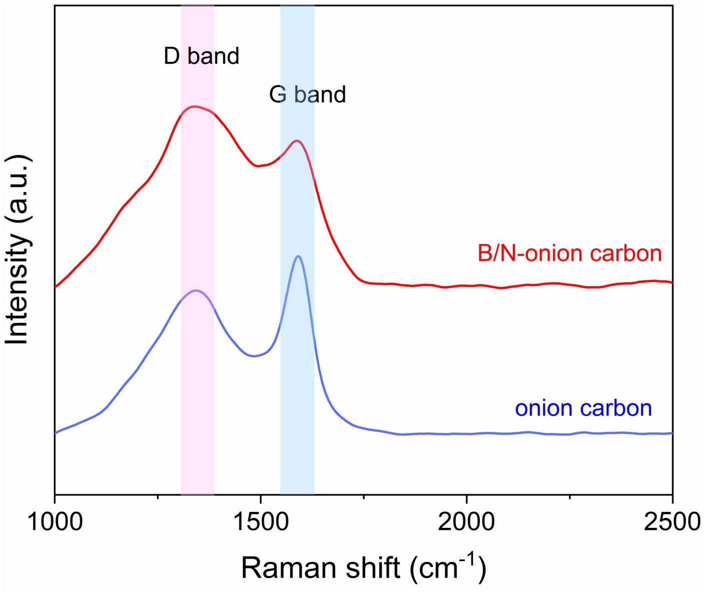

Supplementary Fig. 24 Characterization of onion carbon and B/N-onion carbon. Raman spectra of onion carbon and B/N-onion carbon.

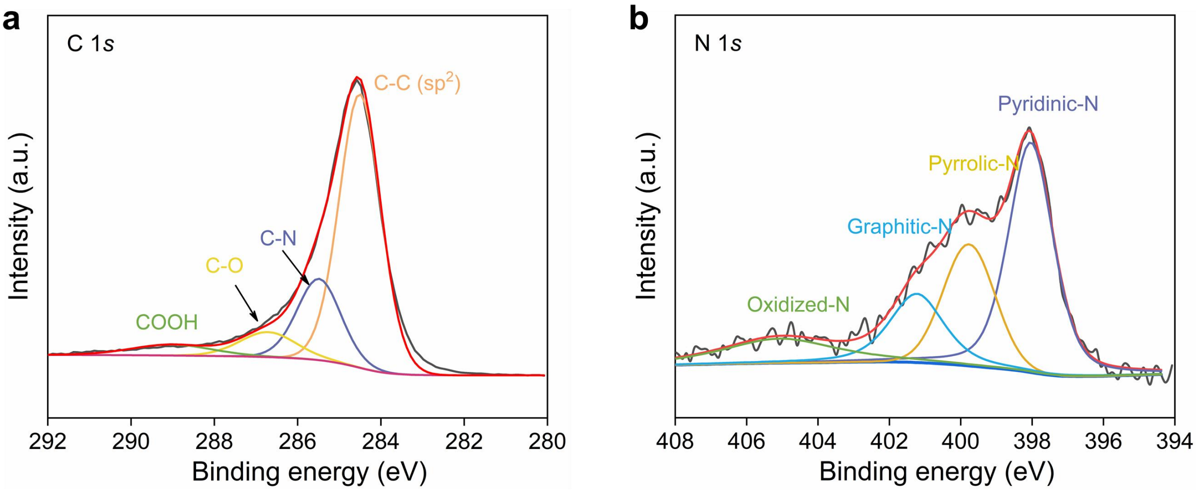

Supplementary Fig. 25 Characterization of N-onion carbon. C 1*s* (**a**) and N 1*s* (**b**) XPS spectra of the N-onion carbon catalyst.

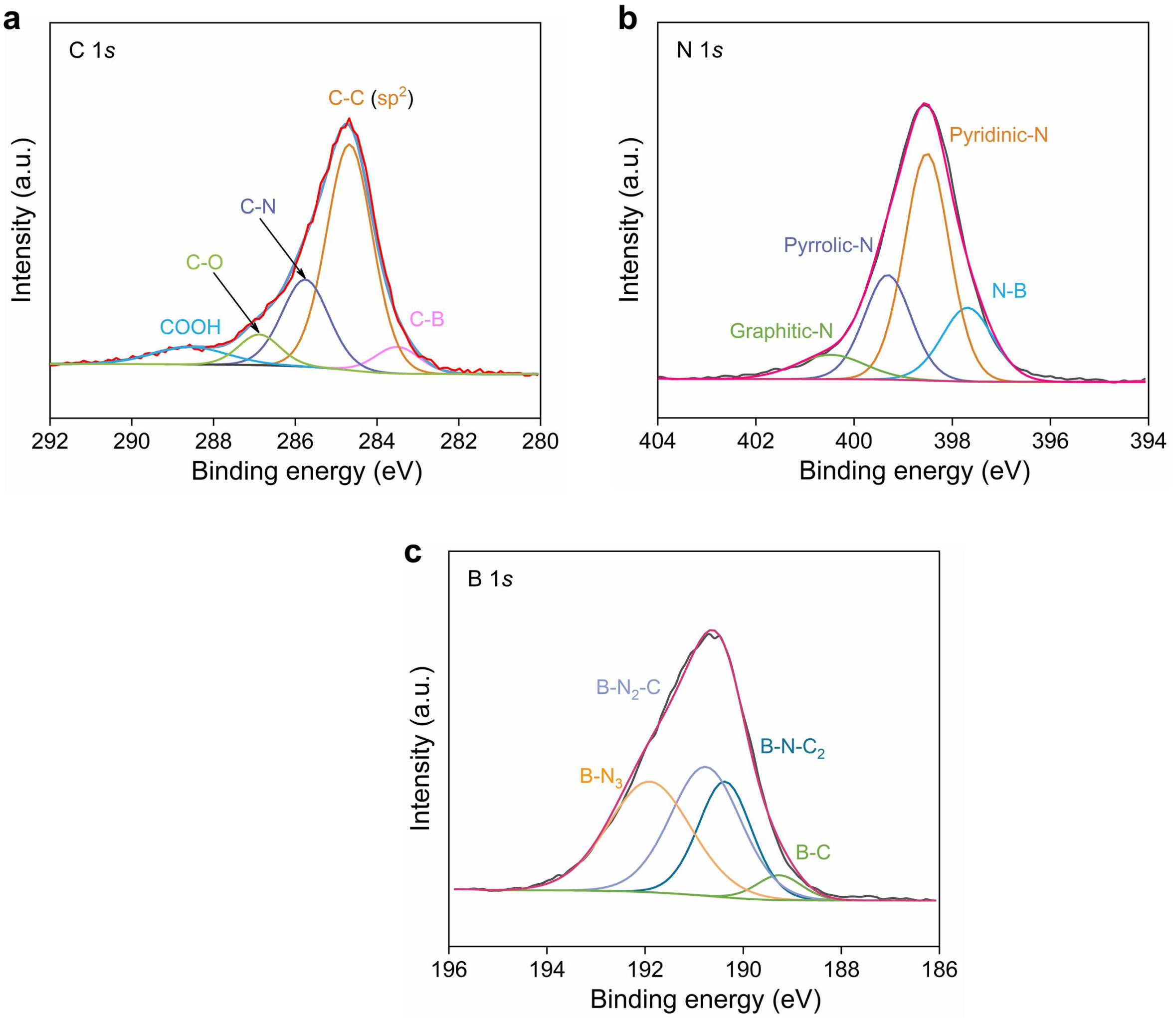

Supplementary Fig. 26 Characterization of B/N-onion carbon. C 1*s* (**a**), N 1*s* (**b**) and B 1*s* XPS spectra of the B/N-onion carbon catalyst.

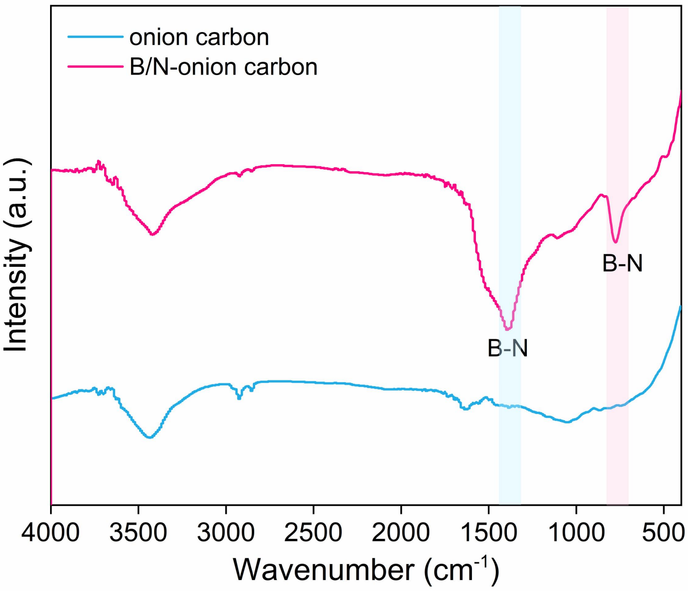

Supplementary Fig. 27 Characterization of B/N-onion carbon. The FTIR spectra of the B/N-onion carbon catalyst.

Performance analysis of cathodic catalysts for H_2O_2 electrosynthesis

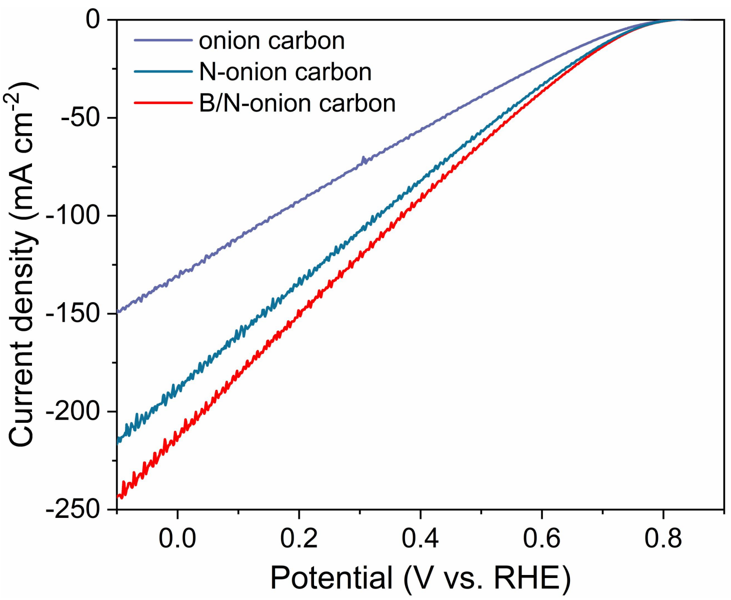

Supplementary Fig. 28 Electrochemical performance of different catalysts for ORR-to-H_2O_2. LSV curves of different catalysts for ORR-to-H_2O_2 were recorded at the scan rate of 20 mV s^{-1} without *iR* compensation. This result matches well with the data provided in Fig. 4e in the main text, suggesting the superior catalysis performance of the B/N-onion carbon.

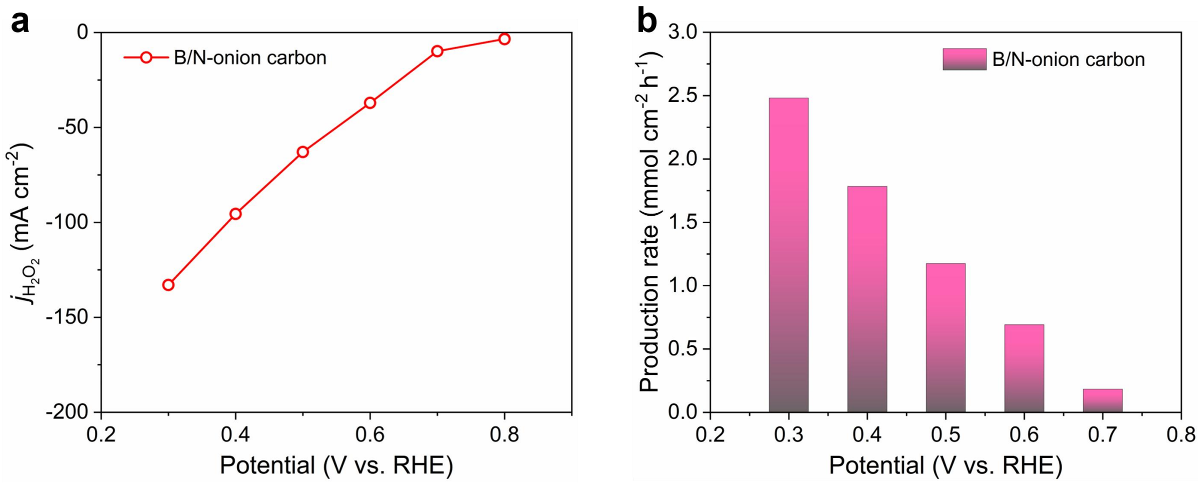

Supplementary Fig. 29 Electrochemical performance of catalysts. a H_2O_2 partial current densities of different catalysts in 1 M KOH without *iR* compensation. **b** H_2O_2 production rates under different applied potentials without *iR* compensation.

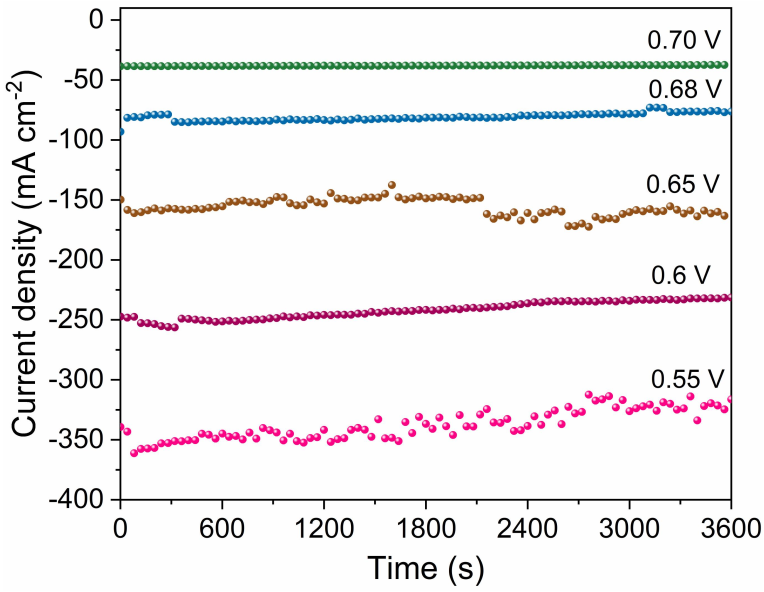

Supplementary Fig. 30 Electrochemical performance of B/N-onion carbon for ORR-to-H_2O_2. Current-time curves of B/N-onion carbon for ORR were recorded under different potentials with 100% *iR* compensation (compensating resistance: 3.1 ± 0.2 Ω).

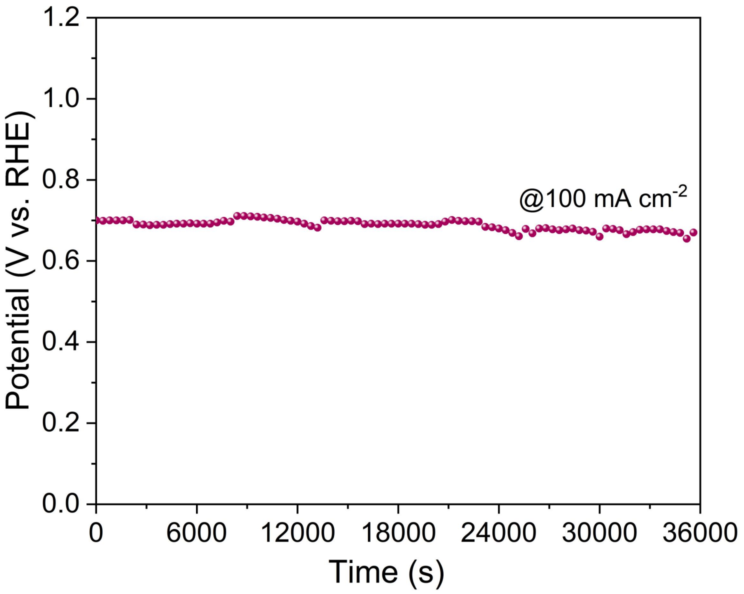

Supplementary Fig. 31 Electrochemical performance of B/N-onion carbon for ORR-to-H_2O_2. The potential-time curve of B/N-onion carbon for ORR-to-H_2O_2 at the current density of 100 mA cm^{-2} with 100% *iR* compensation (compensating resistance: 3.2 ± 0.2 Ω).

Product analysis

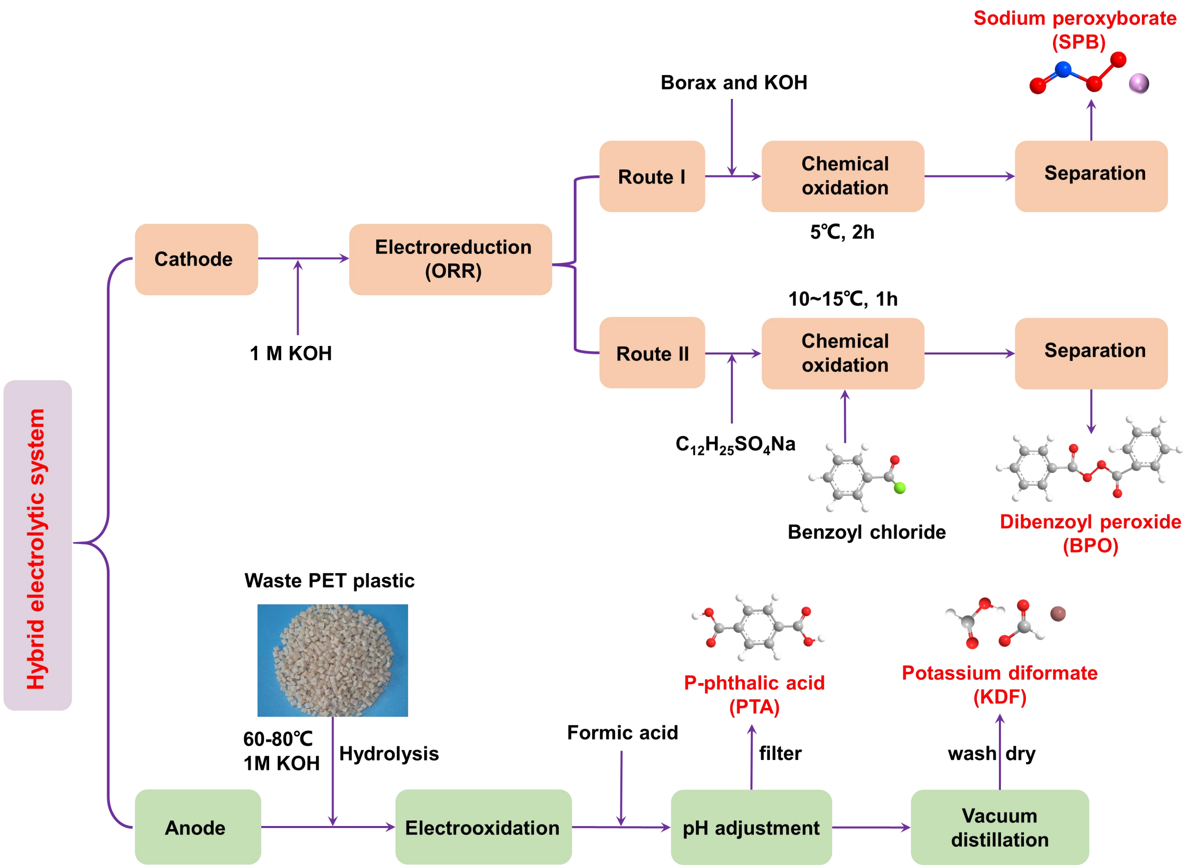

Supplementary Fig. 32 Process design of the hybrid electrolytic configuration. Full process design contains the cathodic oxygen reduction to H_2O_2 system, the anodic PET upcycling system and product separation system. Among them, the cathode product separation system involves two paths. 1) Reaction with borax to prepare downstream sodium peroxyborate (SPB). 2) Reaction with benzoyl chloride to prepare downstream dibenzoyl peroxide (BPO).

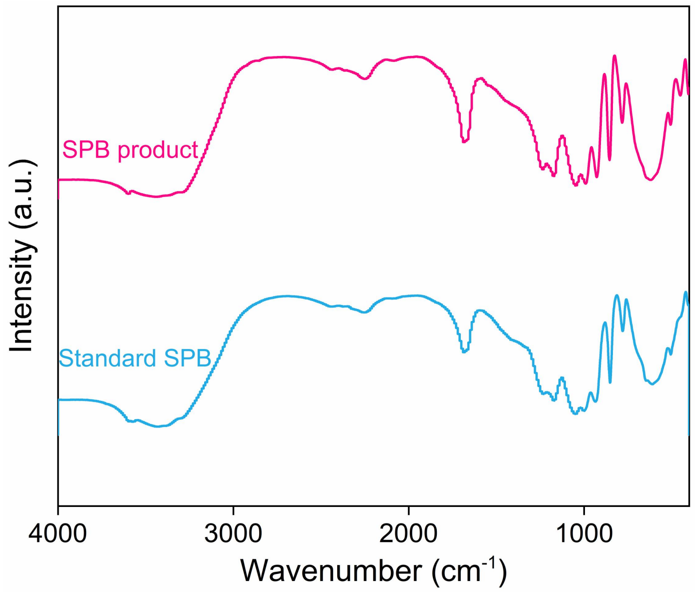

Supplementary Fig. 33 Chemical structural characterization of the SPB product. The FTIR spectra of the SPB product and standard SPB.

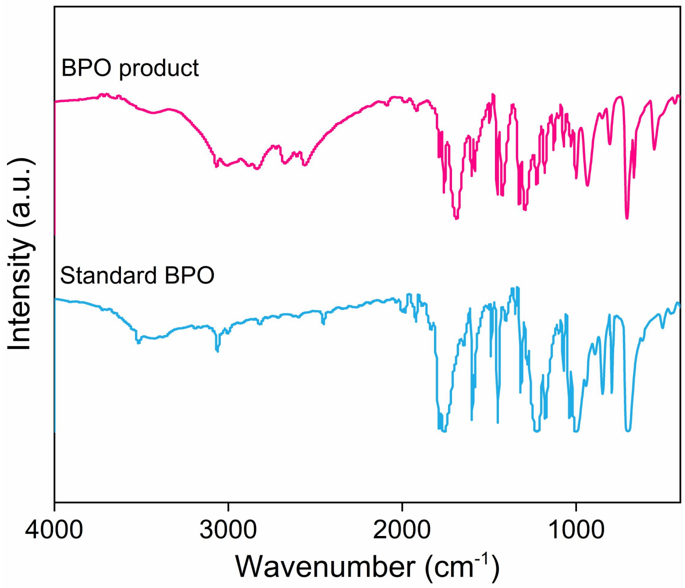

Supplementary Fig. 34 Structural characterization of the BPO product. The FTIR spectra of the BPO product and standard BPO.

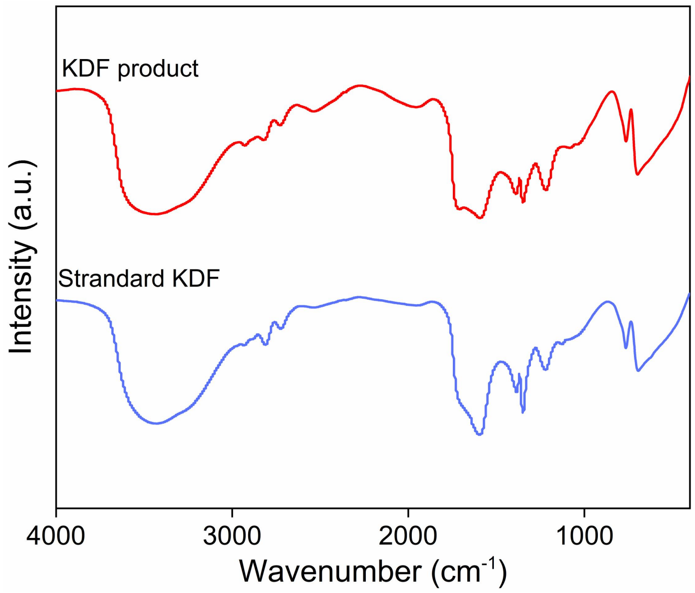

Supplementary Fig. 35 Chemical structural characterization of the KDF product. The FTIR spectra of the KDF product and standard KDF.

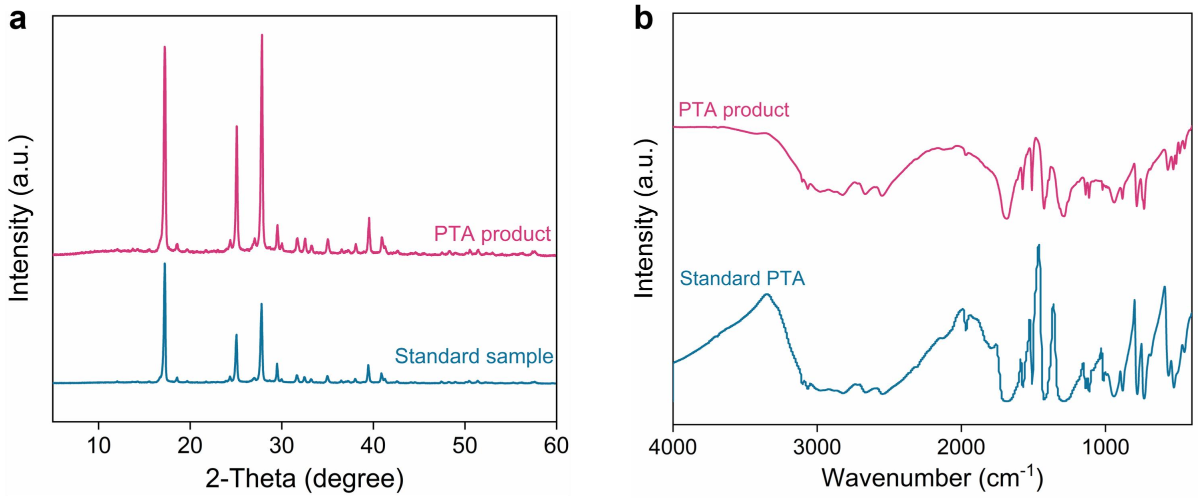

Supplementary Fig. 36 Structural characterizations of the PTA product. a, XRD patterns of the PTA product and standard PTA. **b** The FTIR spectra of the PTA product and standard PTA.

Supplementary Tables

Supplementary Table 1. ICP-OES results of the Ni_1Mn_1-MOF-Se/NF catalyst before and after electrochemical activation.

Activation	Element	Concentration (mg/L)	Content (wt%)
**Before**	**Mn**	0.78	3.60
	**Se**	11.58	56.82
**After**	**Mn**	0.56	2.62
	**Se**	7.68	38.98

Supplementary Table 2. ICP-OES results of Mn and Se elements in the electrolyte during electrochemical activation process and after electrocatalysis test.

Source of electrolyte	Concentration of Mn element (mg/L)	Concentration of Se element (mg/L)
Activation process (0.2 h)	0.28	70.76
Electrocatalysis test (1 h)	0.4	153.93
Electrocatalysis test (3 h)	0.41	155.08

Supplementary Table 3. XPS results of the Ni_1Mn_1-MOF-Se/NF catalyst before and after electrochemical activation.

Activation	Element	Content (Atomic %)
**Before**	**Ni**	13.30
	**Mn**	7.07
	**Se**	17.88
	**O**	21.34
**After**	**Ni**	10.40
	**Mn**	6.45
	**Se**	7.79
	**O**	37.22

Supplementary Table 4. Performance comparison of different electrocatalysts for anodic oxidation.

Catalyst	Electrolyte	Potential (V vs. RHE)	Current density (mA cm^{-2})	Reference
**Ni_1Mn_1-MOF-Se/NF**	1 M KOH + 0.5 M EG	1.36	400	This work
**NiCo**	1 M KOH + 0.1 M Glycerol	1.44	300	Nat. Commun. 2022, 13, 3777
**$Ni_2Fe(CN)_6$**	1 M KOH + 0.33 M Urea	1.40	250	Nat. Energy, 2021, 6, 904-912
**MnCoOOH/NF**	1 M KOH + 0.1 M 1-phenylethanol	1.62	130	Angew. Chem. Int. Ed. 2021, 60, 2-9
**NiB-400**	1 M KOH + 1 M Methanol	1.72	280	Nat. Commun. 2022, 13, 4602
**Ni-Mo-N/CFC**	1 M KOH + 0.1 M Glycerol	1.89	300	Nat. Commun. 2019, 10, 5335
**CoNiCuMnMo/CC**	1 M KOH + 0.1 M Glycerol	1.43	250	J. Am. Chem. Soc. 2022, 144, 7224-7235
**NiMoO-Ar**	1 M KOH + 0.33 M Urea	1.53	300	Energy Environ. Sci., 2018, 11, 1890-1897
**Ni_xB**	1 M KOH + 10 mM HMF	1.46	100	Angew. Chem. Int. Ed. 2018, 57, 11460-11464
**CuONS/CF**	1 M KOH + 1 M Methanol	1.72	320	Angew. Chem. Int. Ed. 2021, 60, 3148-3155
**P-HEOs**	1 M KOH + 10 mM HMF	1.68	30	Angew. Chem. Int. Ed. 2021, 133, 20415-20420
**Au/NiFeOOH**	1 M KOH + 0.1 M BA	1.60	150	Nat. Commun. 2022, 13, 147

Supplementary Table 5. Comparison of electrochemical ORR-to-H_2O_2 performance in flow cell with state of-the-art catalysts.

Catalyst	Electrolyte	Potential (V vs. RHE)	Current density (mA cm^{-2})	High FE (%)	Reference
**B/N-onion carbon**	1 M KOH	0.6	400	97.2	This work
**$N_4Ni_1O_2$/OCNTs**	1 M KOH	0.68	380	96.0	Angew. Chem. Int. Ed. 2021, 61, e202206544
**NiB_2**	0.1 M KOH	0.10	250	93.0	Adv. Mater. 2022, 34, 2202995
**CB-10%**	1 M KOH	0.40	450	95.0	Science 2019, 366, 226-231
**Co-N-C**	0.1 M KOH	--	65	82.0	J. Am. Chem. Soc. 2019, 141, 12372-12381
**VG array**	0.1 M KOH	0.00	200	94.0	Nano Energy 2022, 96, 107046
**Co SA/CC**	0.5 M H_2SO_4	0.00	60	80.0	Carbon Energy 2020, 2, 276-282
**NPC950**	1 M KOH	0.40	100	99.0	ACS Catal. 2021, 11, 13797-13808
**Fe-CNT**	0.1 M PBS	0.35	27	95.0	Nat. Commun. 2019, 10, 3997
**Al_2O_3/PtP_2-600**	H_2O	0.00	160	66.0	Nat. Commun. 2020, 11, 3928
**N-FLG-8**	0.1 M KOH	0.40	45	95.0	Adv. Energy Mater. 2020, 10, 2000789
**NADE**	0.05 M Na_2SO4	--	240	66.8	Nat. Commun. 2020, 11, 1731
**B-C**	1 M KOH	0.67	300	99.0	Nat. Commun. 2021, 12, 2245

Supplementary Table 6. Performance comparison of different electrocatalysts for ORR coupling with anodic oxidation.

Catalyst	Electrolyte	Coupling reaction	Cell voltage (V)	Current density (mA cm^{-2})	Reference
**B/N-onion carbon // Ni_1Mn_1-MOF-Se/NF**	1 M KOH//1 M KOH+0.5 M EG	ORR//EOR	0.79<br>0.93	200<br>400	This work
**B-C//IrO_2/NC**	H_2O	ORR//OER	2.45	400	Nat. Commun. 2021, 12, 2245
**N-FLG-8//Ni foam**	0.1 M KOH+Fu	ORR//FuOR	1.80	120	Adv. Energy Mater. 2020, 10, 2000789
**CMK-3//$Ni_2Fe(CN)_6$**	1 M KOH //1 M KOH + 0.33M Urea	ORR//UOR	0.65	50	Nat. Energy, 2021, 6, 904-912
**CoN4/VG//DSA**	0.1 M $HClO_4$	ORR//OER	1.80	240	Energy Environ. Sci. 2022, 15, 1172-1182
**Co SA/CC//RuO_2**	0.5 M H_2SO_4	ORR//OER	1.60	60	Carbon Energy 2020, 2, 276-282
**NPC950//IrO_2/Ti**	1M KOH//0.5 M H_2SO_4	ORR//OER	1.90	180	ACS Catal. 2021, 11, 13797-13808
**Co-N-C//Ir-MMO/Ti**	0.1 M KOH	ORR//OER	2.00	50	J. Am. Chem. Soc. 2019, 141, 12372-12381
**CB-10%//IrO_2**	0.5 M H_2SO_4	ORR//OER	2.15	200	Science 2019, 366, 226-231
**BP2000//IrO_2**	0.03 M Na_2SO_4	ORR//OER	1.94	100	Nat. Commun. 2022, 13, 2880
**CNB-ZIL 8//NiFe/FeF**	0.1 M KOH	ORR//OER	2.50	133	Angew. Chem. 2022, 134, e202206915

Supplementary Table 7. Summary and comparison for HER coupling anodic oxidation.

Catalyst	Electrolyte	Current density (mA cm^{-2})	Cell voltage (V)	Reference
**$Ni_2Fe(CN)_6$**	1 M KOH + 0.33 M Urea	300	1.60	Nat. Energy, 2021, 6, 904-912
**$NiFeN_x$-NF//$NiFeO_x$-NF**	1 M KOH+0.1 M Glucose	300	1.56	Nat. Commun. 2020, 1, 265
**Ni foam//$Ni(OH)_2$-SDS**	1 M KOH+50 nM Cyclohexanone	140	3.40	Nat. Commun. 2022, 13, 5009
**$CoNi_{0.25}P$/NF**	1 M KOH+0.5 M EG	400	1.73	Nat. Commun. 2021, 12, 4679
**Fe-Ni_3S_2/NF**	1 M KOH + 0.5 M Urea	600	1.78	Adv. Energy Mater. 2022, 2201913
**Ni_2P NPA/NF**	1 M KOH+10 mM HMF	200	1.70	Angew. Chem. Int. Ed. 2016, 55, 9913-9917
**CoNiCuMnMo-NPs/CC**	1 M KOH+0.1 M Glycerol	330	1.81	J. Am. Chem. Soc. 2022, 144, 7224-7235
**NiCo**	1 M KOH+0.1 M Glycerol	120	1.65	Nat. Commun. 2022, 13, 3777
**Ni_3S_2/NF**	1 M KOH+10 mM HMF	100	1.63	J. Am. Chem. Soc. 2016, 138, 13639-13646
**$Co(OH)_2$@$Ni(OH)_2$**	1 M KOH+1 M Ethanol	200	1.58	Energy Environ. Sci., 2022, 10.1039/D2EE01816K
**MoO_2-FeP@C**	1 M KOH+10 mM HMF	100	1.70	Adv. Mater. 2020, 32, 2000455
**Ni-Mo-N-CFC**	1 M KOH+0.1 M Glycerol	90	1.80	Nat. Commun. 2019, 10, 5335
**OMS-Ni_1-CoP.**	1 M KOH+0.5 M EG	70	2.00	Appl. Catal. B-Environ. 2022, 316, 121667
**O-NiMoP/NF**	1 M KOH+0.5 M Urea	150	1.81	Adv. Funct. Mater. 2021, 31, 2104951
**NF/NiMoO-Ar// NF/NiMoO-H_2**	1 M KOH+0.5 M Urea	200	1.67	Energy Environ. Sci. 2018, 11, 1890-1897

Supplementary Table 8. Price of raw materials and products.

Chemicals	Market price (USD/ton)	Source
**Waste PET**	390	Nat. Commun. 2021, 12, 4679
**KOH**	1280	Nat. Commun. 2020, 11, 265
**H_2O**	0.22	Nat. Commun. 2020, 11, 265
**Formic acid**	740	Nat. Catal. 2021, 4, 943-951
**H_2**	1900	Science 2020, 368, 1228-1233
**O_2**	35	Nat. Commun. 2019, 10, 5193
**PTA**	1260	Nat. Commun. 2021, 12, 4679
**KDF**	1590	Nat. Commun. 2021, 12, 4679
**H_2O_2**	1500	Nat. Commun. 2021, 12, 2245
**BPO**	3500	http://www.100ppi.com

Supplementary Table 9. Main parameters in production.

**Parameters**	**data**
**Processing capacity**[3]	150 ton/day
**Operating time**[3]	350 days/yr
**PET impurity content**[3]	10%
**O_2 demand**[7]	75000 kg/day
**Faradaic efficiency of formate from EG**	90%
**Faradaic efficiency of formate from H_2O_2**	93%
**KDF yield**[3]	70%
**PTA yield**[3]	95%
**Equipment replacement cycle**[4, 10]	5 years
**Current density**	300 mA/cm^2
**Electrolyzer area**	1744.9 m^2
**Stack cost**[4]	460 USD/kW
**Electricity price**[2, 4]	0.03 USD/kW h
**Membrane cost**[4]	180 USD/m^2

References

1. Haegel, N. M. et al. Terawatt-scale photovoltaics: Trajectories and challenges. *Science* **356,** 141-143 (2017).
2. Jouny, M., Luc, W. & Jiao, F. General techno-economic analysis of CO_2 electrolysis systems. *Ind. Eng. Chem. Res.* **57,** 2165-2177 (2018).
3. Zhou, H. et al. Electrocatalytic upcycling of polyethylene terephthalate to commodity chemicals and H_2 fuel. *Nat. Commun.* **12,** 4679 (2021).
4. Shin, H., Hansen, K. U. & Jiao, F. Techno-economic assessment of low-temperature carbon dioxide electrolysis. *Nat. Sustain.* **4,** 911-919 (2021).
5. Na, J. et al. General technoeconomic analysis for electrochemical coproduction coupling carbon dioxide reduction with organic oxidation. *Nat. Commun.* **10,** 5193 (2019).
6. Lin, Z. H. et al. Atomic Co decorated free-standing graphene electrode assembly for efficient hydrogen peroxide production in acid. *Energy Environ. Sci.* **15,** 1172-1182 (2022).
7. Xia, Y. et al. Highly active and selective oxygen reduction to H_2O_2 on boron-doped carbon for high production rates. *Nat. Commun.* **12,** 4225 (2021).
8. Li, A. L. et al. Enhancing the stability of cobalt spinel oxide towards sustainable oxygen evolution in acid. *Nat. Catal.* **5,** 109-118 (2022).
9. Zhou, H. et al. Selectively upgrading lignin derivatives to carboxylates through electrochemical oxidative C(OH)-C bond cleavage by a Mn-doped cobalt oxyhydroxide catalyst. *Angew. Chem. Int. Ed.* **60,** 8976-8982 (2021).
10. Verma, S., Lu, S. & Kenis, P. J. A. Co-electrolysis of CO_2 and glycerol as a pathway to carbon chemicals with improved technoeconomics due to low electricity consumption. *Nat. Energy* **4,** 466-474 (2019).
